# Directing the Morphology, Packing, and Properties of Chiral Metal–Organic Frameworks by Cation Exchange**

**DOI:** 10.1002/anie.202205238

**Published:** 2022-06-28

**Authors:** Hadar Nasi, Maria Chiara di Gregorio, Qiang Wen, Linda J. W. Shimon, Ifat Kaplan‐Ashiri, Tatyana Bendikov, Gregory Leitus, Miri Kazes, Dan Oron, Michal Lahav, Milko E. van der Boom

**Affiliations:** ^1^ Department of Molecular Chemistry and Materials Science Weizmann Institute of Science 7610001 Rehovot Israel; ^2^ Department of Chemical Research Support Weizmann Institute of Science 7610001 Rehovot Israel

**Keywords:** Cathodoluminescence, Fluorescence, Magnetic Properties, Metal Cation Metathesis, Metal–Organic Frameworks

## Abstract

We show that metal–organic frameworks, based on tetrahedral pyridyl ligands, can be used as a morphological and structural template to form a series of isostructural crystals having different metal ions and properties. An iterative crystal‐to‐crystal conversion has been demonstrated by consecutive cation exchanges. The primary manganese‐based crystals are characterized by an uncommon space group (*P*622). The packing includes chiral channels that can mediate the cation exchange, as indicated by energy‐dispersive X‐ray spectroscopy on microtome‐sectioned crystals. The observed cation exchange is in excellent agreement with the Irving–Williams series (Mn<Fe<Co<Ni< Cu>Zn) associated with the relative stability of the resulting coordination nodes. Furthermore, we demonstrate how the metal cation controls the optical and magnetic properties. The crystals maintain their morphology, allowing a quantitative comparison of their properties at both the ensemble and single‐crystal level.

## Graphical abstract

Quantitative exchange of metal cations has resulted in a variety of crystals having different optical and magnetic properties but with retention of the crystal shape. Crystals isolated during the exchange exhibited higher concentrations of the incoming metal cations at the hexagonal basal faces, which suggests that the nanochannels are mediating the process.

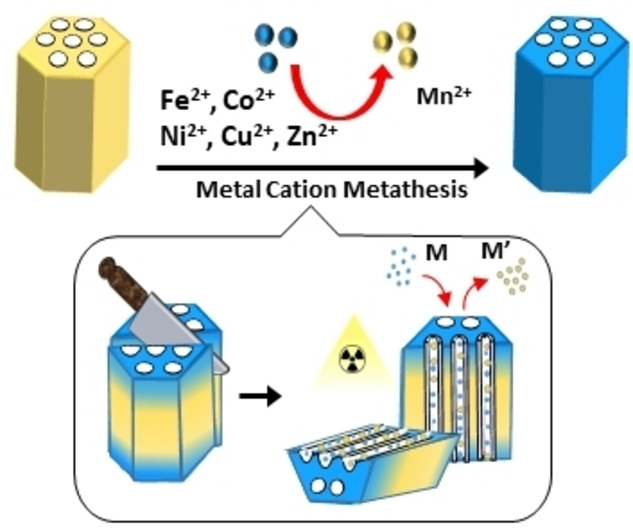

## Introduction

The expression “*single‐crystal to single‐crystal conversion*” refers to a wide range of approaches to modify the atomic nature and/or the structure of materials while preserving their long‐range crystallinity.[^1^, ^2^, ^3^, ^4^] Such post‐synthetic approaches can provide new properties (e.g., optical, magnetic, structural, and mechanical) otherwise not achievable.[^5^, ^6^, ^7^] Some examples include metal cation exchange in semiconductor particles,[^8^, ^9^] quantum dots[^10^, ^11^] and perovskites[^12^, ^13^] as well as galvanic replacement[^14^, ^15^, ^16^] in inorganic materials. Metal–organic frameworks (MOFs) and cages are intriguing materials for single‐crystal to single‐crystal conversion. Their porous nature favors processes of inclusion and exchange of guests (e.g., solvent, molecular guest, and metal ions) in the inner cavities by physical trapping, coordinative interactions with the metal nodes, or pending moieties.[^17^, ^18^, ^19^, ^20^, ^21^, ^22^, ^23^] Moreover, dynamic variations of MOF structures were demonstrated upon exposure to external stimuli[^24^, ^25^, ^26^] and by replacing the constitutive building blocks.[^27^, ^28^, ^29^, ^30^, ^31^] The latter process involves a fine balance between the flexibility and stability of the overall framework. Metal cation metathesis has been mainly demonstrated using a combination of transition metals and linkers having carboxylic acid coordination sites.[^28^, ^32^, ^33^, ^34^, ^35^] Such linkers are classified as hard ligands;^[36]^ they often exhibit planar structures and two or three carboxylic acid groups. For example, Dincă et al. used an exchange of Zn^2+^ with V^2+^ and Ti^3+^ in MOF‐5 to generate crystals that were not obtainable by direct synthesis.^[37]^ A reversible exchange of Cd^2+^ with Pb^2+^ was reported by Kim and co‐workers.^[38]^ However, such exchange processes are less common with MOFs based on metal‐pyridine coordination.[^21^, ^39^, ^40^, ^41^, ^42^] This type of MOF is known to undergo linker exchange due to its moderate ligand field strength.[^43^, ^44^] Nevertheless, it is not obvious that these materials can sustain quantitative metal cation exchanges with the integrity of their frameworks. Competition between selective metal cation exchange and disintegration of the crystal structure is more likely to occur because of the labile nature of such ligands.

**Scheme 1:**
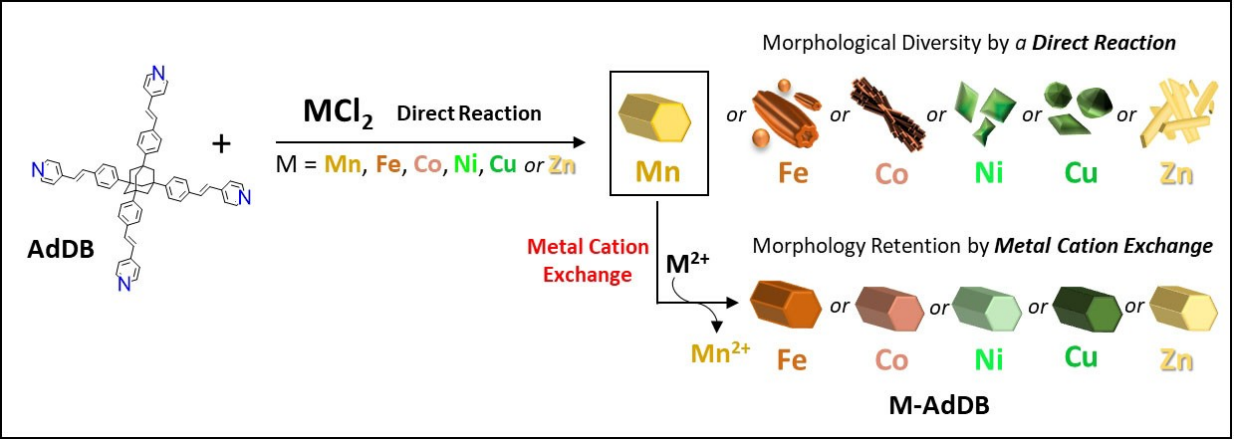
Reaction of the tetrahedral pyridyl ligand, **AdDB**, with divalent first‐row transition metal salts by layering of solvents (top row). The exchange of metal cations, starting from the metal–organic framework (**Mn‐AdDB**), occurs with retention of the morphological uniformity (bottom row).

We have recently introduced a series of MOFs based on tetrahedral pyridyl ligands with divalent cations, including copper and nickel.[^45^, ^46^, ^47^, ^48^] These MOFs have isomorphous crystallographic packing as well as a high level of external uniformity (dimensions and morphology). Their fascinating morphologies can be widely varied by using different experimental parameters (e.g., metal‐to‐ligand ratios, cations, and anions). No additives have been used to direct their morphology and dimensions. To control and predict the packing‐morphology relationship, we investigated cation metathesis using a new, manganese‐based MOF having the same rare *P*622 space‐group as our other reported crystals (Scheme 1).[^45^, ^46^, ^47^, ^48^] The crystal structure is porous and exhibits two geometrically and compositionally different channels. Cation metathesis was used here to control and predict the crystal morphology and elemental composition (from Mn^2+^ to Fe^2+^, Co^2+^, Ni^2+^, Cu^2+^ and Zn^2+^). Interestingly, the porosity of the crystallographic structure facilitates the formation of the new materials. The incoming cations might use the continuous channels running throughout the crystal to reach the nodes. This mechanistic aspect was demonstrated by cutting (microtoming) the crystals and mapping the elemental composition within the structures, namely, the Mn^2+^ to Cu^2+^ exchange, by energy‐dispersive X‐ray spectroscopy (EDS) combined with scanning electron microscopy measurements (SEM). The versatility of the cation metathesis can also be demonstrated by a consecutive Mn^2+^ to Co^2+^ to Cu^2+^ exchange. The direct reaction of the tetrahedral pyridyl ligand with the diverse metal salts, under the same conditions, resulted in different structures; however, by exploiting the single‐crystal to single‐crystal conversion, we generated new MOF structures that preserve their unique crystallographic structures, i.e., they have two different nanochannels. The materials obtained by cation metathesis maintain the hexagonal prism morphology of the starting Mn‐based MOFs. However, their photophysical (absorption, fluorescence, and cathodoluminescence) and their magnetic properties are distinctly different at both the ensemble and single‐crystal level. A quantitative comparison of properties is possible since the effects of shape are essentially factored out.

## Results and Discussion

### Growth of the Primary MOFs

**Figure 1:**
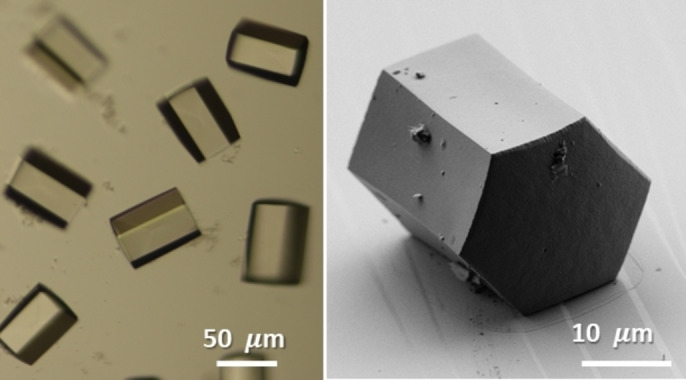
Optical microscope (left) and scanning electron microscope (right) images of **Mn‐AdDB**.

The organic ligand **AdDB**[^49^, ^50^] used here has a tetrahedral (*T*_d_) geometry, an adamantane core, and four phenyl‐vinyl pyridine moieties for coordination to metal cations (Scheme 1). A solution of **AdDB** (1 equiv) in chloroform (1.0 mL) was filtered and added to a glass tube (Ø=10 mm), followed by the addition of methanol (0.5 mL), forming two distinct layers. Then, a solution of MnCl_2_⋅2 H_2_O (3 equiv) in methanol (1.0 mL) was added, forming a third, well‐separated layer. The tube was sealed and tilted (70° from the base) to enlarge the contact areas between the layers. Crystals were observed on the walls after 6 h by the naked eye and allowed to grow for another 42 h. Light microscopy revealed the formation of hexagonal colorless prisms (Figure 1, left). SEM imaging confirmed the structural features of **Mn‐AdDB**, namely, hexagonal prisms having smooth surfaces and sharp edges (width=10–30 μm, length=10–80 μm) (Figure 1, right).

**Figure 2:**
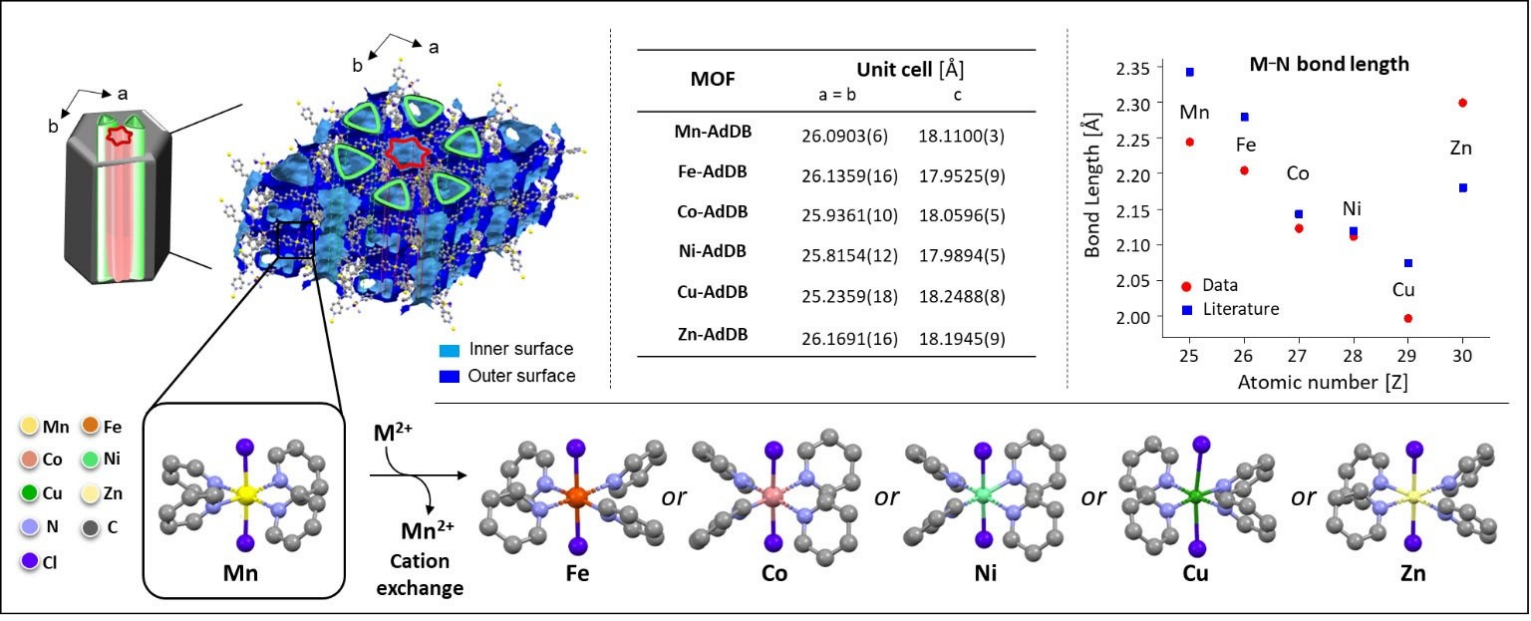
Selected single‐crystal X‐ray diffraction data of **Mn‐AdDB** and the five metal–organic frameworks (MOFs) formed by metal cation exchange (M^2+^=Fe^2+^, Co^2+^, Ni^2+^, Cu^2+^, Zn^2+^). The crystallographic structures have the same hexagonal space group (*P*622). Two helicoidal nanochannels span along the c‐axis with Ø≈0.9 nm (red) and 1.2 nm (green). The unit cell dimensions are listed in the table. The graph summarizes the observed M−N bond lengths of the MOFs (red dots) with literature values (blue square) for the corresponding mononuclear complexes surrounded by monodentate pyridine ligands, i.e., *trans*‐[MCl_2_(pyridine)_4_]^*n*+^.[^59^, ^60^, ^61^, ^62^] Each unit cell contains six propeller‐like coordination nodes with the same handiness. The single‐crystal X‐ray data and structure refinement parameters are summarized in Tables S2–S6 and Figure S1.^[58]^

A single‐crystal X‐ray diffraction (SCXRD) study of **Mn‐AdDB** unambiguously revealed the formation of a MOF (Figures 2, S1 and Table S2). The crystal structure consists of a rarely seen space group *P*622,[^45^, ^46^, ^47^, ^48^, ^51^, ^52^, ^53^, ^54^, ^55^, ^56^, ^57^, ^58^] which is one of the 65 Sohncke groups. This space group indicates chiral packing, although the molecular building blocks are achiral. The divalent metal centers are coordinated to four pyridine moieties of four different ligands and have two Cl anions that form an octahedral geometry. The pyridine moieties are arranged in a “propeller”‐like structure. All six Mn(pyr)_4_ coordination nodes in the unit cell have the same handiness. The Mn−N distances of 2.229(5) Å, 2.245(5) Å, and those of Mn−Cl (2.505(3) Å) are well within the ranges normally found for MnX_2_(pyr)_4_ (X=halide).[^59^, ^60^] Another interesting structural aspect is the presence of two different chiral channels running parallel to the c‐axis and having diameters of 0.9 nm (red) and 1.2 nm (green). The chirality of these channels is evident in the constitutive helicoidally motifs. The inner walls of both channels are formed by helices with the same handiness, making the channels homochiral (Figure S1). The structure contains large solvent accessible voids (39.3 % of the total volume, calculated by Mercury CSD 2020.1.1, employing the contact surface and a spherical probe with a radius of 1.2 Å).

### Metal Cation Exchange

**Figure 3:**
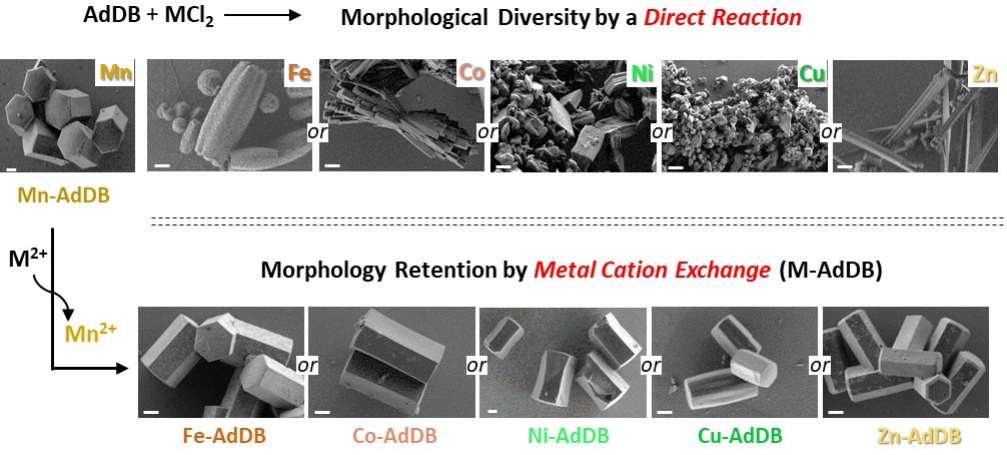
Scanning electron microscope images. Top row: Structures obtained by reacting the tetrahedral pyridyl ligand **AdDB** with metal dichloride salts by layering of solvents. Bottom row: Structures observed after exposing the metal–organic framework (**Mn‐AdDB**) to methanol solutions containing a metal salt. MCl_2_, with M=Fe^2+^, Co^2+^, Ni^2+^, Cu^2+^, Zn^2+^. Scale bar=2 μm.

**Figure 4:**
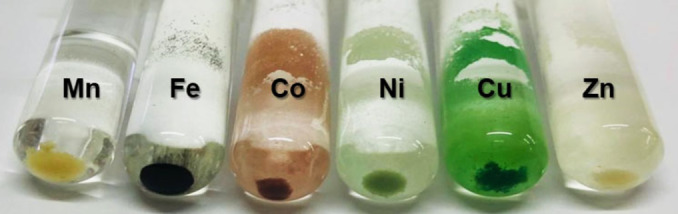
Photograph showing the colors of metal–organic frameworks (MOFs) before (left tube) and after exposing **Mn‐AdDB** to methanol solutions containing a metal salt (MCl_2_, with M=Fe^2+^, Co^2+^, Ni^2+^, Cu^2+^, or Zn^2+^).

Under identical growth conditions for the formation of **Mn‐AdDB**, the use of **AdDB** with other transition metals, namely, FeCl_2_, CoCl_2_, NiCl_2_, CuCl_2_, and ZnCl_2_, resulted in undefined structures, as shown by SEM imaging (Figure 3, top row). Therefore, we used crystals of **Mn‐AdDB** as a primer to exchange the cations while maintaining the morphological uniformity. The mother solution of **Mn‐AdDB** was removed and a methanol solution was added containing one of the abovementioned metal salts. Visually, the bulk materials undergo a clear color change within 48 h, as a first indication of a cation exchange process (Figure 4). Light microscopy revealed i) the preservation of the crystal morphology and dimensions, and ii) the new colors, i.e., green (copper and nickel), orange (iron), and pink (cobalt) (Figure S2). Dissolution and recrystallization processes were not observed by in situ imaging during this time. SEM images confirmed the preservation of the primary prism morphology (Figure 3, bottom and Figure S3). Elemental analysis of **Mn‐AdDB** revealed a composition identical to the formula derived from SCXRD (Table S1). SCXRD measurements and structure refinements showed a crystal‐to‐crystal conversion by metal cation exchange at coordination nodes (Figure 2). The crystallographic packing is retained.^[58]^ The cation exchange of the individual crystals is in agreement with the elemental analysis of the bulk materials that display a quantitative exchange (>99.4 %) of the Mn centers with the cations of the abovementioned salts. The unit cell parameters vary slightly (<3 %). The N_pyr_–metal bond lengths follow the trends reported in the literature for related pyridine complexes.[^60^, ^61^, ^62^, ^63^] All the coordination centers have a distorted octahedral geometry with M−Cl axial distances higher than the equatorial M−N distances. The axial distortion becomes significant for Cu^2+^ (about 2.8 Å) due to the Jahn–Teller effect. The Flack parameters after the cation exchange remain low (Tables S2–S6), indicating that the enantiopurity of the single crystals is preserved.^[64]^ The bulk crystallinity is also preserved after the cation exchange, as indicated by powder X‐ray diffraction measurements and fitting with the SCXRD data (Figure S4). The estimated unit cell parameters are in excellent agreement with the experimental values listed in Figure 2.

We analyzed **Fe‐AdDB** by X‐ray photoelectron spectroscopy (XPS) (Figure S5).No signals indicating the presence of the manganese cations were observed, further confirming that the cation exchange process occurred. Peaks were observed for Fe^3+^ at 711.5 eV (2p_3/2_) and 725.1 eV (2p_1/2_), accompanied by two satellite peaks at ≈719.1 eV and ≈733.5 eV, suggesting the presence of iron oxides on the crystal surface.[^65^, ^66^]

**Figure 5:**
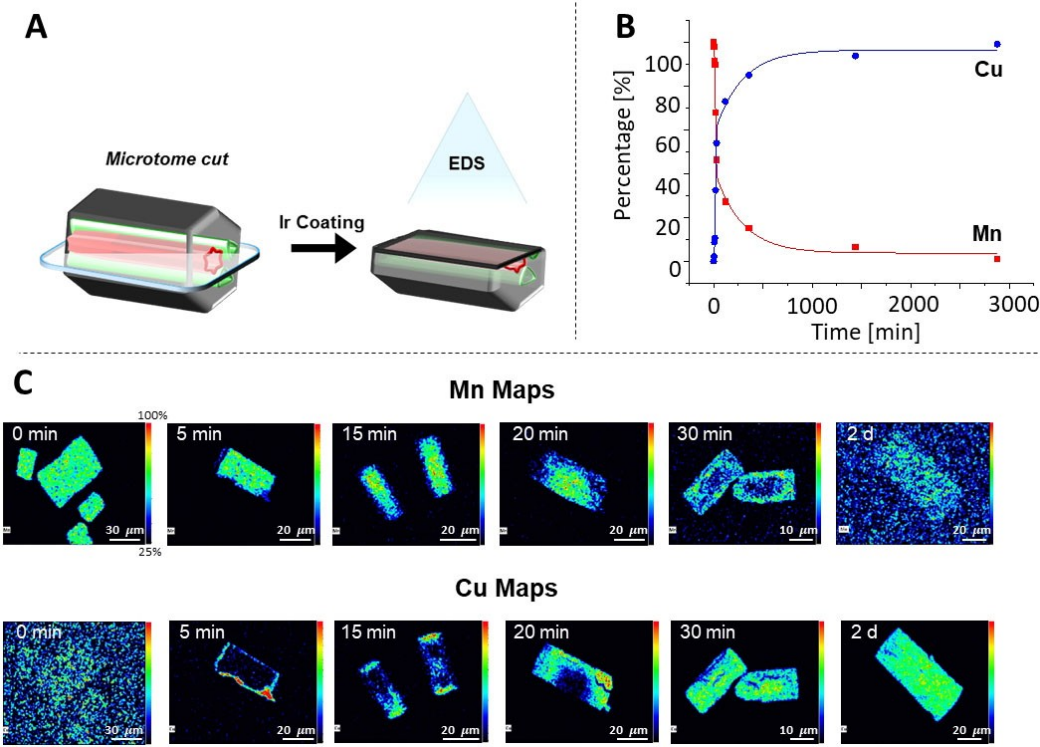
A) Scheme showing the direction of the sectioning of **Mn‐AdDB** by microtome, followed by coating with a thin layer of metallic iridium prior to elemental mapping by energy‐dispersive X‐ray spectroscopy (EDS). B) Graph showing the relative quantities of manganese versus copper inside the crystals as a function of time. C) EDS elemental intensity maps of metal cation content inside the crystals as a function of time.

**Figure 6:**
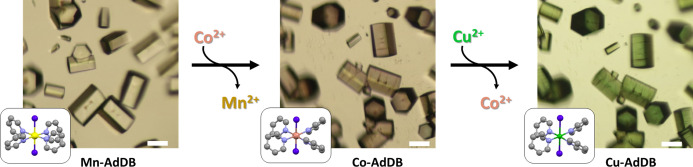
Consecutive cation exchange from **Mn‐AdDB** to **Co‐AdDB**, followed by the formation of **Cu‐AdDB**. The images were obtained by optical microscopy. The insets show the metal coordination nodes from single‐crystal X‐ray diffraction (SCXRD) analysis. Color legend: yellow=Mn; pink=Co; green=Cu, blue=Cl; purple=N; gray=C. Scale bar=50 μm.

**Figure 7:**
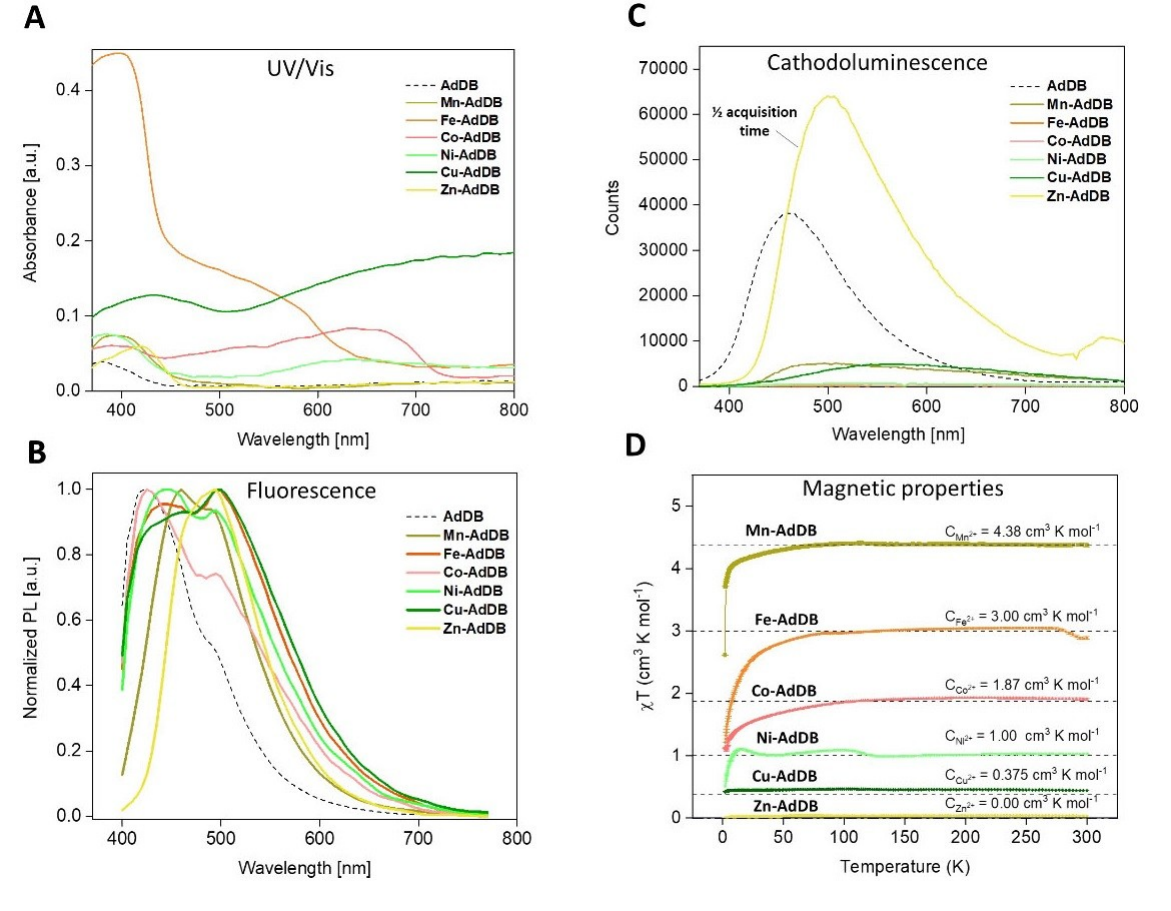
Optical and magnetic properties of **Mn‐AdDB**, **Fe‐AdDB**, **Co‐AdDB**, **Ni‐AdDB**, **Cu‐AdDB**, **Zn‐AdDB**, and **AdDB**. A) Absorbance and B) fluorescence data (*λ*_ex_=355 nm) for bulk samples. C) Cathodoluminescence spectra for single crystals. The same acquisition times were used except for **Zn‐AdDB**. For this MOF, the acquisition time was halved to avoid detector saturation. D) The temperature (*T*) dependence of magnetic susceptibility (*χ*) shown as plots *χ* *T* vs. *T*. All samples were measured at 0.5 T (where T is Tesla) except for **Zn‐AdDB**, which was measured at 2.5 T. The dashed lines denote the theoretical values of the Curie constants for each metal cation. Note the agreement between the theoretical and experimental plots.

The Mn‐to‐Cu exchange was followed *in‐situ* by light microscopy (Figure S6) and by *ex‐situ* SEM, combined with EDS measurements over 2 days. Immediately upon the addition of a light green solution containing CuCl_2_ to **Mn‐AdDB**, the colorless crystals turned green, as observed by the naked eye, along with a concurrent decrease in the color of the solution. No additional color changes were evident after 6 hours. Relatively dark bands were observed parallel to the bases. Similar observations were made for the Mn‐to‐Fe and Mn‐to‐Co exchange. These darker areas are probably due to light scattered by surface irregularities. Such surface features are also indicated by SEM and light microscopy images (Figure S7). The elemental composition inside the crystals during the cation exchange was determined by time‐dependent SEM‐EDS analysis (Figure 5). Samples of the crystals on a silicon wafer were sliced with a microtome to observe exchange processes inside the materials. Then, the cut crystals were covered with a thin layer of iridium to eliminate the charging effects. The spatial information provided by color maps revealed that the Cu^2+^ cations accumulate at all the crystal faces (*t*=5 min). Then, Cu^2+^ mainly enters the crystals from the hexagonal basal {001} faces, with a coinciding decrease in the amount of Mn^2+^ (*t*=15–20 min). Concurrently, the Mn^2+^ ions seem to diffuse from the center of the crystals to the hexagonal {001} faces. After two days, only traces of Mn^2+^ remain. The overall process indicates that the channels direct the metal ion diffusion processes. EDS spectra of the cut crystals provided quantitative information about the elemental composition. Full cation exchange was observed after ≈2 days, with *t*_1/2_≈30 min. We also demonstrated consecutive cation exchange from **Mn‐AdDB** to **Co‐AdDB** and subsequently from **Co‐AdDB** to **Cu‐AdDB** (Figure 6). This experiment was performed by replacing the solutions containing the metal salts. The complete exchange for both steps was confirmed by SCXRD and light microscopy. *In‐situ* light microscopy showed intact crystals that changed color from colorless to pink to green, as expected for the presence of Mn, Co, or Cu, respectively. These experiments show that both morphology and crystal packing are preserved even upon multiple metal exchanges. Our observations show that the metal cation exchange can be used to predict the morphology and structure of our MOFs. The question we address here is how the nature of the metal cation controls the optical and magnetic properties of these materials. We analyzed the properties of a series of MOFs having identical morphologies and similar topologies in the solid state, using both bulk (ensemble) and single‐crystal measurements. Bulk samples were used for the absorption, fluorescence and magnetic measurements (Figure 7A, B, D). Cathodoluminescence was carried out on single crystals that were identified by SEM imaging (Figure 7C, S8). Absolute absorption measurements were performed using an integrating sphere on dried samples with equal weights (Figure 7A). We noted that in this configuration it is possible to directly compare the properties of different compositions because the crystal structure, geometry, and size are similar. It is known that these parameters affect both scattering and reabsorption within a sample. The two MOFs with the longest M‐pyridine coordination bonds, **Mn‐AdDB** and **Zn‐AdDB** (Figure 2), have similar UV/Vis spectra with a single absorption band at *λ*_max_=398 nm and *λ*_max_=421 nm, respectively. These absorption bands are red shifted in comparison with **AdDB**, and they might include its absorption (n–π* and π–π* transitions),[^67^, ^68^] and a contribution from a charge transfer band. The UV/Vis spectra of **Fe‐AdDB**, **Co‐AdDB**, **Ni‐AdDB**, and **Cu‐AdDB** exhibit, along with a similar band at *λ*≈400 nm, strikingly different additional broad bands, most likely due to charge transfer band absorption. This effect is responsible for the dramatically different colors shown in Figure 4. Such charge transfer bands are expected for octahedral complexes.[^69^, ^70^] The widths of these bands increase for shorter M‐pyridine bonds (in agreement with the Irving–Williams series).[^60^, ^61^] The trend of the intensities of the bands in the near IR are **Co‐AdDB**<**Fe‐AdDB**≈**Ni‐AdDB**<**Cu‐AdDB**. For **Ni‐AdDB** and **Cu‐AdDB**, the near IR bands are comparable and higher, respectively, than the absorptions in the UV/Vis range. Such deviations for Ni‐ and Cu‐based MOFs, compared to analogous mononuclear complexes, can be explained as follows. It is known that the extinction coefficients of the *d*–*d* transitions of the Ni^2+^ and Cu^2+^ complexes increase upon distortion of the symmetry around the metal centers.[^70^, ^71^, ^72^, ^73^, ^74^] This observation is in good agreement with distortions in the axial positions of the MOFs, as demonstrated by the single‐crystal X‐ray measurements (Figure 2). Although we cannot be sure about the nature of the charge transfer, absorptions in the visible and near‐IR range have also been attributed to strong metal‐to‐ligand charge transfer (MLCT) contributions by others.^[75]^

The differences in the fluorescence spectra of the MOFs (Figure 7B) are not as dramatic as those observed in the absorption spectra. All the emission spectra seem to be composed of two emission peaks (at ≈420 nm and ≈510 nm) but at different ratios. Notably, both peaks are visible as vibronic transitions in the neat **AdDB** sample. The emission from **Mn‐AdDB** and **Zn‐AdDB** (having long M‐pyridine bonds) spans from ≈400 nm to 650 nm. The emissions of **Co‐AdDB**, **Ni‐AdDB**, **Fe‐AdDB**, and **Cu‐AdDB** all exhibit a charge‐transfer character in their absorption spectra; this extends further to the red, ranging from 400 nm to 700 nm and they exhibit broader emission peaks (FWHMs of 142, 153 nm, 169 nm, and 177 nm, respectively).^[76]^ The quantum yields (QY) of all MOFs are relatively low (<5 %) except for **Zn‐AdDB**, for which the QY, upon excitation at *λ*_ex_=430 nm, is 18±3 % and at *λ*_ex_=440 nm it is 27±3 % (Table S7).

Indeed, Zn‐based MOFs have been shown to exhibit some of the highest QYs in any such system, with record values exceeding 90 %.[^77^, ^78^] These QYs are related to the *d* ^10^ electronic state of the metal center.^[79]^ The fully occupied *d* orbitals of the Zn centers do not allow for ligand‐to‐metal charge transfer, thus isolating the fluorescent ligands. In addition, processes related to fluorescence‐quenching absorption are excluded.[^80^, ^81^] Interestingly, the shift between the emission peak of **AdDB** and **Zn‐AdDB** is very large, ≈70 nm, much more than the characteristic values of <15 nm.^[77]^ This shift most likely occurs because **Zn‐AdDB** preferentially emits in the redder vibronic band. This effect might be caused by a change in the overlap between the excited electronic state of the ligand and the higher vibrational bands in the ground state induced by ligand deformation. Fluorescence‐lifetime measurements show that the emission of **Zn‐AdDB** can be assigned to the ligand, in good agreement with the above‐discussed QYs (Figure S9). The **Mn‐AdDB** lifetime is within the system response function of ≈0.3 nsec. Longer and similar lifetimes of ≈0.8 nsec at the 500 nm emission peak for both the **AdDB** and the **Zn‐AdDB** were observed, supporting the isolation of the fluorescent ligands. In addition, lifetimes at the 425 nm emission peak are similar for all samples and are within the system response function. The emission properties of single crystals were also measured by SEM‐cathodoluminescence imaging microscopy (Figure 7C, S8). The emission spectra induced by cathodoluminescence clearly differ from those induced by optical excitation. The MOFs exhibit single and broad bands ranging from 400 to 800 nm and have maxima between 480 and 570 nm. In addition, a distinct band was observed at 784 nm for **Zn‐AdDB**. These bands are likely associated with the formation of charged species due to the electron irradiation. In agreement with the fluorescence measurements of the bulk (Figure 7B), the strongest signal was observed for **Zn‐AdDB**. The metal cation exchange also resulted in MOFs having distinctly different magnetic properties. The MOFs with magnetically active cations, **Mn‐AdDB**, **Fe‐AdDB**, **Co‐AdDB**, **Ni‐AdDB**, and **Cu‐AdDB**, show linear dependences of the magnetic moment as a function of the applied magnetic field at 300 K (Figure S10A). At 5 K, the magnetic moment approaches a plateau, as described for paramagnets by the Brillouin function (Figure S10B).[^82^, ^83^] For both temperatures, no hysteresis was observed. Plots of *χ* *T* vs *T*, where *χ* is the magnetic susceptibility of the samples, are shown in Figure 7D. At sufficiently high temperatures, when the energy of thermal motion prevails over the energy of the exchange interactions, the values of *χ* *T* approach the theoretical values of the Curie constant (*C*) for the used metal bivalent cations,[^81^, ^82^, ^83^, ^84^, ^85^] as is expected by the Curie–Weiss law. The *χ* *T* value increases as a function of the cation Cu^2+^<Ni^2+^<Co^2+^<Fe^2+^<Mn^2+^. **Zn‐AdDB** is a weak diamagnetic.

## Conclusion

We have demonstrated that pyridine‐based MOFs formed by tetrahedral linkers are suitable for quantitative metal cation exchange, with retention of both their crystallinity and morphology. The exchange process is accompanied by changes in the optical and magnetic properties of the crystals. The use of tetratopic linkers for such single‐crystal to single‐crystal conversion is rare. An example of metal cation exchange with a tetratopic and planar carbocyclic acid‐based ligand was recently reported by Kaskel and co‐workers.^[86]^ However, tetrahedral linkers, as reported in this study, have not been used. Such non‐planar ligands are especially interesting because of their propensity to form highly complex and chiral structures that contain continuous channels.[^45^, ^46^, ^47^, ^48^, ^87^] Although all facets of the crystals are accessible to the incoming metal cations, the exchange process proceeds along the direction of these channels. This observation might indicate that the channels facilitate the diffusion of the metal cations in and out of the crystal structure. The crystal structure analysis of the initial crystals revealed coordinatively saturated metal centers, strongly indicating that the exchange processes involve pyridine‐metal dissociation steps prior to the exchange processes. Although speculative, the role of defects cannot be excluded.^[57]^ Binding of incoming cations might induce stress that results in destabilization of adjacent metal‐coordination sites. Such proposed dynamic behavior is more likely to occur with pyridine linkers that have a moderate ligand field strength than with hard ligands such as carboxylic acids.^[36]^ Engineering of the optical properties of MOFs can be achieved by anion exchange,^[88]^ ligand modifications,[^89^, ^90^, ^91^, ^92^] doping MOFs with metal ions, and metal ion inclusions.[^88^, ^93^, ^94^, ^95^] In our MOFs, the different metal cations strongly affect the electronic and vibronic properties reflected in ligand‐to‐metal and metal‐to‐ligand charge transfer contributions and notable changes in the fluorescence properties. The absorbance varies from low‐absorbing MOFs in a narrow UV/Vis window to crystals having strong absorptions spanning the whole visible and even the near‐IR range. Notably, the fact that the crystals maintain their morphology greatly facilitates a quantitative comparison of their optical properties, as measured at both the ensemble and single‐crystal level, since the effects of shape are essentially factored out. Furthermore, we have shown that the cation exchange resulted in both para‐ and diamagnetic MOFs. The crystals are potentially useful for energy harvesting and optical switches.

## Conflict of interest

The authors declare no conflict of interest.

1

## Supporting information

As a service to our authors and readers, this journal provides supporting information supplied by the authors. Such materials are peer reviewed and may be re‐organized for online delivery, but are not copy‐edited or typeset. Technical support issues arising from supporting information (other than missing files) should be addressed to the authors.

Supporting Information

Supporting Information

Supporting Information

Supporting Information

Supporting Information

Supporting Information

Supporting Information

Supporting Information

Supporting Information

Supporting Information

Supporting Information

Supporting Information

## Data Availability

The data that support the findings of this study are openly available in ChemRxiv at https://doi.org/10.26434/chemrxiv‐2021‐brq1h.
