# Supplementary material for: Directing the Morphology, Packing, and Properties of Chiral Metal–Organic Frameworks by Cation Exchange
Source: Angew Chem Int Ed Engl. 2022 Jun 28;61(34):e202205238. doi: 10.1002/anie.202205238 (PMC9542332; doi:10.1002/anie.202205238)
Supplement: Supplementary file 1 — Supporting Information [file ANIE-61-0-s006.pdf]

Angewandte Chemie
Eine Zeitschrift der Gesellschaft Deutscher Chemiker

Supporting Information

Directing the Morphology, Packing, and Properties of Chiral Metal–Organic Frameworks by Cation Exchange

H. Nasi, M. Chiara di Gregorio, Q. Wen, L. J. W. Shimon, I. Kaplan-Ashiri, T. Bendikov, G. Leitus, M. Kazes, D. Oron, M. Lahav*, M. E. van der Boom**

Table of Contents

Methods	S2
Experimental Section	S4
Supplementary Tables	S5
Supplementary Figures	S12
References	S22

Methods

Light microscopy. Optical images were taken using a Nikon microscope Eclipse E600 Pol.

Scanning electron microscopy (SEM). SEM measurements were performed using HRSEM Zeiss ULTRA-55 and HRSEM Zeiss SUPRA-55 VP instruments using a landing voltage of 2 kV. SEM samples were prepared by placing a drop of the reaction mixture on a silicon substrate and drying under air.

Microtome Sectioning. Crystals were drop-casted on a silicon substrate and the solvent was allowed to evaporate. An Eppendorf tube (0.5 mL) was filled with Lowicryl HM20 resin (Electron Microscopy Sciences, USA); then the substrate was placed in the tube with the crystals facing towards the resin. The tubes were placed upside-down (to ensure an efficient embedding of the crystals in the resin) in an AFS2 freeze substitution device (Leica Microsystems, Vienna Austria) and were treated for 48 h at -20 °C, for 12 h at 0 °C and 12 h at room temperature. The samples were sectioned with a diamond knife (Diatome, Biel, Switzerland) using a UC7 ultramicrotome (Leica Microsystems, Vienna Austria).

Energy dispersive X-ray spectroscopy (EDS). EDS measurements were performed using a four-quadrant detector (Bruker QUANTAX FlatQUAD) installed on the Zeiss Ultra 55. The elemental analysis was done with and without an iridium coating (2-3 nm-thick, using plasma sputtering, by Safematic CCU-010 HV coater) at a landing voltage of 5/10 kV. Imaging of secondary electrons was acquired with a Everhart-Thornley detector.

X-ray photoelectron spectroscopy (XPS). XPS measurements were carried out with a Kratos AXIS ULTRA system using a monochromatic Al Kα X-ray source (hν = 1486.6 eV) at 75W and detection pass energies ranging between 20 and 80 eV. A low-energy electron flood gun (eFG) was applied for charge neutralization. Curve fitting analysis was based on linear or Shirley background subtraction and application of Gaussian-Lorenzian line shapes. The reaction mixtures were drop-casted on silicon wafers.

Elemental analysis. Elemental analyses (C, H, N, Cl, Mn, Fe, Co, Ni, Cu and Zn) were performed at Kolbe Laboratorium, Mulheim, Germany. The oxygen is calculated as the leak to 100%. The error is ±0.01% for C, H, N; ±0.015% for Cl; ±0.015% for Mn in **Mn-AdDB**, ±0.015% for Fe; ±0.00075% for Mn in **Fe-AdDB**, ±0.02% for Co; ±0.005% for Mn in **Co-AdDB**, ±0.025% for Ni; ±0.0005% for Mn in **Ni-AdDB**, ±0.01% for Cu; ±0.0005% for Mn in **Cu-AdDB**, and ±0.01% for C, H, and N; ±0.02% for Cl; ±0.015% for Mn and Zn in **Zn-AdDB**. The crystals were collected into an Eppendorf tube and dried in vacuum (2 μbar) at room temperature (24 h).

Powder X-Ray Diffraction (PXRD). PXRD measurements were performed by reflection geometry using an Ultima III (Rigaku, Japan) diffractometer equipped with a sealed Cu anode X-ray tube operating at 40 kV and 40 mA. The crystalline powders, along with their mother solutions, were withdrawn from the test tube by a pipette and dispersed on an aluminum holder. The holder was placed in the chamber of the instrument. Subsequently, the chamber was under vacuum and the temperature was gradually reduced by liquid nitrogen. A bent graphite monochromator and a scintillation detector were aligned to the diffracted beam. Next, $\theta/2\theta$ scans were performed under specular conditions in the Bragg–Brentano mode with variable slits. The samples were scanned from 2 to 22 degrees in step mode with a step size of 0.025 degrees and a collection time of 1 degree/min. The XRD patterns were analyzed and fitted using Jade Pro 2010 software (Materials Data, Inc.).

Single-Crystal X-Ray Diffraction (SCXRD). Hexagonal prism colorless crystals (**Mn-AdDB**, **Table S2**), pink crystals (**Co-AdDB**, **Table S4**), light green crystals (**Ni-AdDB**, **Table S4**), green crystals (**Cu-AdDB**, **Table S3**), red crystals (**Fe-AdDB**, **Table S5**), colorless crystals (**Zn-AdDB**, **Table S5**), pink crystals (**Co-AdDB**, **Table S6**), and green crystals (**Cu-AdDB**, **Table S6**) for used for single-crystal X-ray analyses. For **Mn-AdDB** and **Co-AdDB**, two crystals from two different batches were analyzed. Data for three crystals of **Cu-AdDB** from three different batches were collected. The crystals were flash frozen in the liquid nitrogen gaseous stream of an

OxfordSystems Cryosystem. Diffraction data for the Mn, Cu, Ni, and Co systems were collected with CuKα1 λ = 1.54184 Å measured on either a RigakuOD XtaLab^{pro} X-ray diffractometer equipped with a Detris PilatusR 200K-A detector or on a Rigaku Synergy R with a HyPic-Arc 150°. The diffraction data for the Fe, Zn, and Co systems were collected with MoKα λ = 0.71073 measured on a Rigaku XtaLab Synergy diffractometer with a Pilatus 300K CdTe detector. The crystals were kept at 100K. Data were collected as ω scans of 0.5º frames with CrysAlisPro. The data were integrated and reduced using CrysAlisPro (Rigaku 2018). An absorption correction (either gaussian or multi-scan) was applied. The structures were solved by direct methods using SHELXT-2016/4[S1], as implemented in Olex2.[S2] The structures were fully refined with SHELXL-2016/4[S1] All non-hydrogen atoms were refined with anisotropic displacement coefficients. Hydrogen atoms were placed in calculated positions and assigned isotropic displacement coefficients, and their coordinates were allowed to ride on the respective carbon atoms. The SQUEEZE protocol of Platon or the solvent masking routine of Olex2 was used.[S3] Alternative SCXRD refinement of the structures with Mn (no exchange) or Mn/M mixed occupancy metal sites (partial exchange) by allowing for the free refinement of metal composition occupancy (total occupancy was constrained to 1) was also examined. The resulting refinement R factors, goodness-of-fit, and the refinement stability of the structures were used to determine the final metal composition. For details, see **Table S2** and the cif files **v486** and **v395**, **Table S3** for cif files **v445** and **v510**, **Table S4** for cif files **v508** and **v509**, **Table S5** for cif files **v428-sq** and **v432**, and **Table S6** for cif files **v430** and **v433b**.

Fluorescence spectroscopy. The crystals were drop-casted from methanol suspensions onto glass slides. The ligand (**AdDB**) was deposited by smearing. Next, the glass slides were placed vertically in the beam pathway. The sample was excited by a frequency tripled Nd:YAG Q-switched laser, pumping an optical parametric oscillator (Ekspla NT342/C/3/UVE), with a pulse duration of 5 nsec and a repetition rate of 10 Hz. The fluorescence spectra were collected at ~30º using a 20×0.4 NA objective, spectrally filtered using a color glass filter and a monochromator (Acton SpectraPro2150i) and were measured by a photomultiplier tube (Hamamatsu R10699). The photomultiplier transient output was measured by a 600 MHz digital oscilloscope (LeCroy Wavesurfer 62Xs). The laser beam pulse energy was measured by a pyroelectric sensor (PE9-C, Ophir Optronics). Lifetime measurements were measured as the above except that a 355 nm, <0.5 nsec Teem Photonics laser and a R5108 Hamamatsu PMT were used.

Measuring magnetic properties using a Superconducting Quantum Interference Device (SQUID). The measurements were carried out with a SQUID magnetometer MPMS3 (LOT-Quantum Design, Inc.) using the vibrating sample magnetometry (VSM) mode, applying a peak amplitude of 6 mm with a frequency of 13 Hz, and an average time of 5 s. The samples were mounted on a standard brass holder. Plots of the magnetic moments as a function of the applied magnetic field at constant temperatures of 5K and 300K are shown in Figure S9. The magnetic field was applied in intervals of T≤H≤+6T in both directions (H = magnetic field). Measurements of χT vs T, where χ is the magnetic susceptibility of the samples, were performed in the temperature range of 2K-300K while applying a magnetic field of 0.5T (**Mn-AdDB**, **Fe-AdDB**, **Co-AdDB**, **Ni-AdDB**, **Cu-AdDB**), and 2.5 T (**Zn-AdDB**). The measurements were carried out using the field-cooled mode: briefly, the sample was cooled from room temperature in the same field that was used for measurements during the subsequent heating. The temperature dependencies of the magnetic susceptibility were normalized to moles using the chemical formula: $C_{62}H_{52}MN_4Cl_2$, were M = Mn^{2+}, Fe^{2+}, Co^{2+}, Ni^{2+}, Cu^{2+} or Zn^{2+}. In the χT vs T plot, in order to separate the paramagnetic contribution from the molar magnetic susceptibility, we fitted the temperature dependencies by a Curie-Weiss (CW) equation in the form: $\chi^{mol} = C/(T-\theta) + \chi_0$, where C = Curie constant, θ = Weiss parameter, χ_0 = contribution of diamagnetism from the orbital motion of electrons (temperature independent), and the samples impurities and holder. The dotted lines in Figure 7D, indicating the Curie constant values, were calculated: $C = N_A\mu^2_B / 3k_B\, g^2S(S+1)$, where N_A = Avagadro number, k_B = Boltzmann constant, μ_B = Bohr magneton, S = spin quantum number of correspondent ion and g is the factor Lande taken as g = 2.

Cathodoluminescence Microscopy. Scanning electron microscopy (SEM), combined with cathodoluminescence (CL) spectra, was collected using a Gatan MonoCL4 Elite system equipped with a retractable diamond-turned mirror. The collected light was first imaged in panchromatic mode using a high-sensitivity PMT (photomultiplier tube) with a spectral range of λ = 160-930 nm. The collected light was then directed to a monochromator and a charge-coupled device (CCD) for parallel spectroscopy. The spectral range was set to λ = 300-800 nm with a band pass of 20 nm by choosing the 150 lines/mm grating centered on 550 nm and a 1 mm entrance slit. The CL system is installed on a Zeiss Gemini SEM 500, a high-resolution SEM equipped with a two-mode field emission gun. CL measurements were performed at 5kV with an aperture of 60 μm, and with high current in analytical gun mode. First, light and electron images were collected simultaneously. Then, CL spectra were collected on defined spots (pixel size 560 nm) that were marked on the pre-scanned SEM image. The acquisition time per spot was set to 20 sec for **AdDB**, **Mn-AdDB**, **Fe-AdDB**, **Co-AdDB**, **Ni-AdDB**, and **Cu-AdDB** and 10 sec for **Zn-AdDB** (Figure S8). Spectra were collected on several crystals.

Solid-state UV spectra. UV absorbance spectra and the absolute PL quantum yield were collected by a HAMAMATUS Absolute PL Quantum Yield Spectrometer C11347 with a wavelength range of λ = 370-850 nm. The instrument is equipped with a 150 W Xenon light source and an integrating sphere consisting of a 3.3 inch Spectralon. The samples were drop-casted from methanol solutions on a quartz petri dish and left to dry at room temperature. The samples were weighed before the measurements. The measurements were conducted between λ = 370 to 800 nm with intervals of 10 nm. The quantum yields are reported in Table S7 for **Zn-AdDB** and **AdDB**.

Experimental Section

Materials and Methods. 1-Bromoadmantane (99%), benzene (99.7%), $AlCl_3$, iodine, bis(triphenylphosphine)palladium(II)dichloride, 4-vinylpyridine (95%), $CuCl_2$ (97%), $CoCl_2{\cdot}6H_2O$ (98%), and N-methyl-2-pyrrolidone (NMP) were purchased from Sigma Aldrich. The following compounds were also purchased: t-butylbromide (Apollo), bis(trifluoroacetoxy)iodo)benzene (Fluka Chemika), chloroform ($CHCl_3$, ≥ 99.8%) (Bio-Lab), triethylamine (Alfa Aesar), diethyl ether (Bio-Lab), dimethylformamide (DMF, ≥ 99.8%) (Alfa Aesar), $FeCl_2{\cdot}4H_2O$ (98%) (Alfa Aesar), MnCl2·2H2O (Merck, ≥ 99%), $NiCl_2{\cdot}6H_2O$ (97%) (BDH – laboratory reagents), and $ZnCl_2$ (98%) (Fluka). Reagents were used without further purification. Glass pressure tubes (Ace Glass, Inc., pressure tubes #15 with a plunger valve, PTFE Bushing and FETFE® O-Ring, volume 50 mL) were cleaned by immersion in a base bath (1.5 M, NaOH) for 2 to 3 days, then washed with water and ethanol. The preparation of 1,3,5,7-tetrakis{4-[(E)-2-pyridine-4-yl-vinyl]phenyl}admantane (**AdDB**) was carried out according to a literature procedure.[S5,S6]

Preparation of Mn-AdDB. The crystals were obtained by layering solvents in a tube of borosilicate glass (⌀ = 10 × 75 mm, 4 mL volume) at room temperature. A solution of **AdDB** (3.5 mg, 4.1 μmol, 3.5 mg/mL, 1 eq) in chloroform (1.0 mL) was filtered using cotton and injected into the tube. A layer of methanol (0.5 mL) was added on top of the chloroform. Then, a layer of $MnCl_2{\cdot}2H_2O$ (2.0 mg, 12.3 μmol, 2.0 mg/mL, 3 eq) in methanol (1.0 mL) was placed on the layer of methanol. The tube was sealed, tilted (70° from the base) without mixing the solvents, to enlarge the contact areas between the layers by ~3✕ in order to facilitate the diffusion process. The formation of colorless hexagon crystals was observed after 6 h by a light micro-scope (length: 10–80 μm, width: 10–30 μm). The crystals were left in the mother solution at room temperature. The yield of **Mn-AdDB** was 48%.

Direct preparation of Fe-AdDB', Co-AdDB', Ni-AdDB', Cu-AdDB', and Zn-AdDB'. The samples were obtained by layering solvents in a borosilicate glass tube (⌀ = 10 × 75 mm, 4 mL) at room temperature. For **Fe-AdDB'**, a solution of **AdDB** (4.5 mg, 5.3 μmol, 4.5 mg/mL, 1 eq) in chlo-roform (1.0 mL) was filtered using cotton and injected into the tube. A layer of methanol (0.5 mL) was added on top of the layer of chloroform. Then, a layer of the metal salt, $FeCl_2{\cdot}4H_2O$ (2.0 mg, 15.8 μmol, 2.0 mg/mL, 3 eq) in methanol (1.0 mL), was placed on top of the layer of methanol. The tube was sealed and tilted (70° from the base) to enlarge the contact areas between the layers, in order to facilitate the diffusion process. After 6 h, an orange powder appeared on the tube wall. The same procedure was used for forming the other crystals. **Co-AdDB'**: **AdDB** (2.4 mg, 2.8 μmol, 2.4 mg/mL, 1 eq), $CoCl_2{\cdot}6H_2O$ (2.0 mg, 8.4 μmol, 2.0 mg/mL, and 3 eq). **Ni-AdDB': AdDB** (2.4 mg, 2.8 μmol, 2.4 mg/mL, and 1 eq), $NiCl_2{\cdot}6H_2O$ (2.0 mg, 8.4 μmol, 2.0 mg/mL, and 3 eq). **Cu-AdDB'**: **AdDB** (4.2 mg, 4.9 μmol, 4.2 mg/mL, and 1 eq), $CuCl_2$ (2.0 mg, 14.8 μmol, 2.0 mg/mL, and 3 eq). **Zn-AdDB'**: **AdDB** (3.3 mg, 3.9 μmol, 3.3 mg/mL, and 1 eq), $ZnCl_2$ (2.0 mg, 14.6 μmol, 2.0 mg/mL, and 3 eq) were used. The resulting materials have the following colors: **Co-AdDB'**, pink; **Ni-AdDB'**, light green; **Cu-AdDB'**, green; and **Zn-AdDB'** colorless.

Crystal-to-Crystal Conversion by Exchange of Mn^{2+} by Fe^{2+}, Co^{2+}, Ni^{2+}, Cu^{2+}, and Zn^{2+}. After the formation of **Mn-AdDB**, the mother liquid was removed with a syringe from the tube. Subsequently, a freshly prepared solution of $FeCl_2{\cdot}4H_2O$ (1.6 g, 8.2 mmol, and 400 mg/mL), $CoCl_2{\cdot}6H_2O$ (1.9 g, 8.2 mmol, and 485 mg/mL), $NiCl_2{\cdot}6H_2O$ (1.9 g, 8.2 mmol, and 484 mg/mL), $CuCl_2$ (15 mg, 0.11 mmol, and 3.8 mg/mL), or $ZnCl_2$ (15.6 mg, 1.15 mmol, and 3.9 mg/mL) in methanol (4.0 mL) was slowly added to the tube. The tube was sealed by Parafilm, tilted (70° from the base), and left at room temperature for two days. Subsequently, the solution was removed by a syringe. The colorless crystals left in a solution containing $CuCl_2$ gradually became green. For the crystals immersed in solutions containing $FeCl_2{\cdot}4H_2O$, $CoCl_2{\cdot}6H_2O$, and $NiCl_2{\cdot}6H_2O$, the intense color of the solutions prevented us from observing changes in the color of the crystals during the experiment. After isolation, the crystals appeared orange ($FeCl_2{\cdot}4H_2O$), pink ($CoCl_2{\cdot}6H_2O$), and light green ($NiCl_2{\cdot}6H_2O$). The crystals remained colorless after the Mn^{2+} to Zn^{2+} exchange. Finally, the crystals were isolated, washed with methanol (4✕), and were stable at room temperature in methanol for six months.

Consecutive Crystal-to-Crystal Conversion by Metal Cation Exchange of Mn^{2+} by Co^{2+}, followed by the Exchange of Co^{2+} by Cu^{2+}. The mother liquid of **Mn-AdDB** was removed with a syringe from the glass tube. Subsequently, a freshly prepared solution of $CoCl_2{\cdot}6H_2O$ (1.9 g, 8.2 mmol, 485 mg/mL) in methanol (4.0 mL) was slowly added to the tube. The tube was sealed by Parafilm, tilted (70° from the base), and left at room temperature for 2 days. Subsequently, the solution was removed by a sy-ringe. Then, the crystals were washed four times using methanol. This step concludes the first metal exchange process; pink crystals of **Co-AdDB** were formed, as shown by SCXRD. The same sample of **Co-AdDB** was then immersed in a solution of $CuCl_2$ (52.1 mg, 8.2 mmol, and 13.0 mg/mL) in methanol (4.0 mL). The tube was sealed by Parafilm, tilted (~70° from the base), and left at room temperature for 2 days. The sample underwent identical solvent removal and crystal washing processes as those previously described for the first metal exchanging process. This step concluded the second metal exchange process; green crystals of **Cu-AdDB** were formed, as shown by light microscopy and SCXRD.

Table S1. Element weight percentage and the crystal formula obtained from elemental analysis of the bulk samples of the crystals.

	% C	% H	% N	% Mn	% M	% Cl	% O	Formula
**Mn-AdDB** 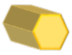	75.53	5.34	5.67	5.79	-	7.56	4.26	$[C_{62}H_{52}Cl_{2.1}MnN_4]$
**Fe-AdDB** 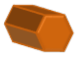	68.06	4.72	4.74	0.05	9.16	11.69	1.58	$[C_{62}H_{51}Cl_{3.6}Fe_{1.8}N_{3.7}O]$
**Co-AdDB** 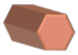	74.0	5.52	5.93	<0.01	5.79	7.04	1.72	$[C_{62}H_{55}Cl_2CoN_4O]$
**Ni-AdDB** 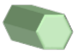	61.18	6.11	4.75	0.54	10.14	15.97	1.31	$[C_{62}H_{73.8}Cl_{5.5}Ni_{2.1}N_4O]$
**Cu-AdDB** 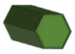	72.61	5.17	5.56	0.06	7.10	7.94	4.82	$[C_{62}H_{53}Cl_{2.3}Cu_{1.2}N_{4.1}O]$
**Zn-AdDB** 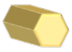	66.77	4.81	5.32	1.06	8.23	11.05	2.76	$[C_{62}H_{53.2}Cl_{3.5}Zn_{1.4}N_{4.2}O_{1.9}]$

Table S2. Single-crystal X-ray data and the structure refinement parameters for **Mn-AdDB**.

Crystal	**Mn-AdDB** 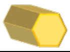	**Mn-AdDB** 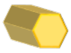
Measurement	V486	V395
CCDC	2153577	2024344
Diffractometer	Rigaku XtaLab Synergy R, HyPix-Arc 150	Rigaku XtaLab[Pro]
Empirical formula	$C_{62}H_{52}Cl_2MnN_4$ + [solvent]	$C_{62}H_{52}Cl_2MnN_4$ + [solvent]
Formula weight (g/mol)	978.92	978.91
Temperature (K)	100	100
Wavelength (Å)	1.54184	1.54184
Crystal system	hexagonal	hexagonal
Space group	*P622*	*P622*
Unit cell dimensions	a = 26.0903(6) Å α = 90°<br>b = 26.0903(6) Å β = 90°<br>c = 18.1100(3) Å γ = 120°	a = 26.1251(6) Å α = 90°<br>b = 26.1251(6) Å β = 90°<br>c = 18.1734(3) Å γ = 120°
Volume (Å^3)	10676.0(5)	10741.9(5)
Z	6	6
Density calculated (g/cm^3)	0.914	0.908
Absorption coefficient (mm^{-1})	2.438	2.423
F(000)	3066	3066
Theta range for data collection (°)	3.9130 to 70.4940	3.908 to 58.934
Index range	$-24 \leq h \leq 30$<br>$-31 \leq k \leq 29$<br>$-21 \leq l \leq 19$	$-24 \leq h \leq 24$<br>$-28 \leq k \leq 16$<br>$-20 \leq l \leq 18$
Reflection collected (unique)	40500 (6540)	24845 (5164)
R_{int}	0.0582	0.0375
Completeness %	99.9	99.5
Data/restraints/parameters	6540 /35/ 326	5164 / 101 / 302
Goodness-of-fit on F^2	1.017	1.046
Final *R* [I>2σ(I)]	R_1 = 0.0782, wR_2 = 0.2271	R_1 = 0.0736, wR_2 = 0.2248
R_1, wR_2	R_1 = 0.0985, wR_2 = 0.2480	R_1 = 0.0829, wR_2 = 0.2348
Largest diff. peak and hole (e. Å^{-3})	0.658 / -0.297	0.487 / -0.304
Flack Parameter	0.108(15)	0.04(2)
Hexahedral (inner layer) and triangular channels handiness	*M*	*P*
Hexahedral (outer layer)	*P*	*M*

Table S3. Single-crystal X-ray data and structure refinement parameters after 2 days of exchange by $CuCl_2$ at room temperature, **Cu-AdDB**.

Crystal	**Cu-AdDB** 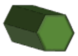	**Cu-AdDB** 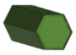
Measurement	V445	V519
CCDC	2078788	2153550
Diffractometer	Rigaku XtaLab^{Pro}	Rigaku XtaLab Synergy R, HyPix-Arc 150
Empirical formula	6($C_{62}H_{52}Cu_{0.75}Mn_{0.25}N_4Cl_2$) +[solvent]	$C_{62}H_{52}Cl_2CuN_4$ + [solvent]
Formula weight (g/mol)	987.53	987.51
Temperature (K)	100	100
Wavelength (Å)	1.54184	1.54184
Crystal system	hexagonal	hexagonal
Space group	*P*622	*P*622
Unit cell dimensions	a = 25.393(2) Å α = 90°	a = 25.2359(18) Å α = 90°
	b = 25.393(2) Å β = 90°	b = 25.2359(18) Å β = 90°
	c = 18.234(2) Å γ = 120°	c = 18.2488(8) Å γ = 120°
Volume (Å^3)	10182.2(12)	10064.7(15)
Z	1	6
Density calculated (g/cm^3)	0.966	0.978
Absorption coefficient (mm^{-1})	1.429	1.446
F(000)	3090	3090
Theta range for data collection (°)	3.48 to 68.196	3.503 to 68.235
Index range	$-26 \leq h \leq 30$<br>$-29 \leq k \leq 23$<br>$-19 \leq l \leq 21$	$-23 \leq h \leq 30$<br>$-27 \leq k \leq 26$<br>$-21 \leq l \leq 21$
Reflection collected (unique)	27347 (6238)	40085 (6185)
R_{int}	0.0390	0.0561
Completeness %	99.9	99.9
Data/restraints/parameters	6238 / 32 / 315	6185 / 66 / 325
Goodness-of-fit on F^2	0.96	0.902
Final *R* [I>2σ(I)]	R_1 = 0.0616, wR_2 = 0.1823	R_1 = 0.0753, wR_2 = 0.2126
R_1, wR_2	R_1 = 0.0891, wR_2 = 0.2059	R_1 = 0.1280 wR_2 = 0.2585
Largest diff. peak and hole (e. Å^{-3})	0.419 / -0.264	0.441 / -0.203
Flack Parameter	0.13(5)	0.05(2)
Hexahedral (inner layer) and triangular channels handiness	*M*	*M*
Hexahedral (outer layer)	*P*	*P*

SUPPORTING INFORMATION

Table S4. Single-crystal X-ray data and structure refinement parameters after 2 days of exchange by $NiCl_2$ and $CoCl_2$ at room temperature for **Ni-AdDB** and **Co-AdDB**.

Crystal	**Ni-AdDB** 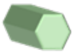	**Co-AdDB** 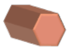
Measurement	V508	V517
CCDC	2153548	2153549
Diffractometer	Rigaku XtaLab Synergy R, HyPix-Arc 150	Rigaku XtaLab Synergy R, HyPix-Arc 150
Empirical formula	$C_{62}H_{52}Cl_2NiN_4$ + [solvent]	$C_{62}H_{52}Cl_2CoN_4$ + [solvent]
Formula weight (g/mol)	982.68	982.91
Temperature (K)	100	100
Wavelength (Å)	1.54184	1.54184
Crystal system	hexagonal	hexagonal
Space group	*P622*	*P622*
Unit cell dimensions	a = 25.8154(12) Å α = 90°	A = 25.9361(10) Å α = 90°
	b = 25.8154(12) Å β = 90°	b = 25.9361(10) Å β = 90°
	c = 17.9894(5) Å γ = 120°	c = 18.0596(5) Å γ = 120°
Volume (Å^3)	10382.6(10)	10520.8(9)
Z	6	6
Density calculated (g/cm^3)	0.943	0.931
Absorption coefficient (mm^{-1})	1.360	2.862
F(000)	3084.0	3078.0
Theta range for data collection (°)	3.937 to 65.074	3.9080 to 74.0710
Index range	$-31 \leq h \leq 31$<br>$-30 \leq k \leq 21$<br>$-15 \leq l \leq 21$	$-32 \leq h \leq 22$<br>$-16 \leq k \leq 30$<br>$-22 \leq l \leq 19$
Reflection collected (unique)	29094 (6810)	29325 (6936)
R_{int}	0.0258	0.0330
Completeness %	96.5	96.6
Data/restraints/parameters	6810 / 13 / 332	6936 / 5 / 331
Goodness-of-fit on F^2	0.970	1.081
Final *R* [I>2σ(I)]	R_1 = 0.0891, wR_2 = 0.2059	R_1 = 0.1137, wR_2 = 0.2798
R_1, wR_2	R_1 = 0.0616, wR_2 = 0.1823	R_1 = 0.0828, wR_2 = 0.2502
Largest diff. peak and hole (e. Å^{-3})	0.399 / -0.203	1.185 / -0.357
Flack Parameter	0.03(3)	0.022(11)
Hexahedral (inner layer) and triangular channels handiness	*P*	*P*
Hexahedral (outer layer)	*M*	*M*

Table S5. Single-crystal X-ray data and structure refinement parameters after 2 days of exchange by $FeCl_2$ and $ZnCl_2$ at room temperature for **Fe-AdDB** and **Zn-AdDB**.

Crystal	**Fe-AdDB** 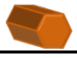	**Zn-AdDB** 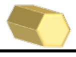
Measurement	v428-sq	v432
CCDC	2078970	2078971
Diffractometer	Rigaku XtaLab Synergy S	Rigaku XtaLab Synergy S
Empirical formula	$C_{62}H_{52}Cl_2FeN_4$ + [solvent]	$C_{62}H_{52}Cl_2ZnN4$ + [solvent]
Formula weight (g/mol)	979.88	989.43
Temperature (K)	100	100
Wavelength (Å)	0.71073	0.71073
Crystal system	hexagonal	hexagonal
Space group	*P622*	*P622*
Unit cell dimensions	a = 26.136(2) Å α = 90°	a = 26.169(2) Å α = 90°
	b = 26.136(2) Å β = 90°	b = 26.169(2) Å β = 90°
	c = 17.9525(9) Å γ = 120°	c = 18.1945(9) Å γ = 120°
Volume (Å^3)	10620.1(11)	10790.7(11)
Z	6	6
Density calculated (g/cm^3)	0.919	0.9135
Absorption coefficient (mm^{-1})	0.320	0.447
F(000)	3076.9	3100.6
Theta range for data collection (°)	1.8 to 27.1	1.8 to 25.3
Index range	$-28 \le h \le 33$<br>$-33 \le k \le 33$<br>$-23 \le l \le 21$	$-27 \le h \le 26$<br>$-15 \le k \le 15$<br>$-21 \le l \le 21$
Reflection collected (unique)	42505 (7851)	50432 (6604)
R_{int}	0.0436	0.0669
Completeness %	99.8	99.9
Data/restraints/para-meters	7831 / 57 / 332	6604 / 57 / 325
Goodness-of-fit on F^2	0.961	0.994
Final *R* [I>2σ(I)]	R_1 = 0.0671, wR_2 = 0.1907	R_1 = 0.0695, wR_2 = 0.2057
R_1, wR_2	R_1 = 0.0957, wR_2 = 0.2088	R_1 = 0.1049, wR_2 = 0.2348
Largest diff. peak and hole (e. Å^{-3})	0.50 / -0.41	0.48 / -0.62
Flack Parameter	0.04(3)	0.22(9)
Hexahedral (inner layer) and triangular channels handiness	*M*	*M*
Hexahedral (outer layer)	*P*	*P*

Table S6. Single-crystal X-ray data and structure refinement parameters after 2 days of metal cation exchange from **Mn-AdDB** to **Co-AdDB**. **Cu-AdDB** was obtained after 2 days of metal cation exchange from **Co-AdDB**.

Crystal	**Co-AdDB** 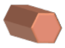		**Cu-AdDB** 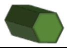	
Measurement	v430b		v433b	
CCDC	2169064		2078972	
Diffractometer	Rigaku XtaLab Synergy S		Rigaku XtaLab^{Pro}	
Empirical formula	$C_{62}H_{52}Cl_2CoN_4$ + [solvent]		$C_{62}H_{52}Cl_2CuN_4$ + [solvent]	
Formula weight (g/mol)	982.90		987.51	
Temperature (K)	100		100	
Wavelength (Å)	0.71073		1.54184	
Crystal system	hexagonal		hexagonal	
Space group	*P*622		*P*622	
Unit cell dimensions	a = 26.0185(15) Å	α = 90°	a = 25.4448(6) Å	α = 90°
	b = 26.0185(15) Å	β = 90°	b = 25.4448(6) Å	β = 90°
	c = 18.1354(11) Å	γ = 120°	c = 18.2223(3) Å	γ = 120°
Volume (Å^3)	10632.2(14)		10217.2(5)	
Z	6		6	
Density calculated (g/cm^3)	0.921		0.963	
Absorption coefficient (mm^{-1})	0.349		1.424	
F(000)	3078.0		3090.0	
Theta range for data collection (°)	1.927 to 27.103		3.147 to 77.366	
Index range	$-28 \leq h \leq 33$<br>$-33 \leq k \leq 27$<br>$-16 \leq l \leq 23$		$-28 \leq h \leq 32$<br>$-27 \leq k \leq 23$<br>$-21 \leq l \leq 22$	
Reflection collected (unique)	43858 (7860)		28213 (7037)	
R_{int}	0.0447		0.0309	
Completeness %	99.8		97.8	
Data/restraints/parameters	7860 / 65 / 283		7037 / 0 / 321	
Goodness-of-fit on F^2	1.058		1.055	
Final *R* [I>2σ(I)]	R_1 = 0.0706 wR_2 = 0.2080		R_1 =0.0615, wR_2 = 0.1859	
R_1, wR_2	R_1 = 0.1039, wR_2 = 0.2351		R_1 = 0.0748, wR_2 = 0.2035	
Largest diff. peak and hole (e. Å^{-3})	0.469 / -0.236		0.53 / -0.23	
Flack Parameter	0.024(11)		0.07(4)	
Hexahedral (inner layer) and triangular channels handiness	*P*		*M*	
Hexahedral (outer layer)	*M*		*P*	

Table S7. Quantum yields for **Zn-AdDB** and **AdDB**.

Wavelength [nm]	Quantum Yield Zn-AdDB	Quantum Yield AdDB
370	0.117	0.309
380	0.089	0.206
390	0.092	0.092
400	0.102	0.281
410	0.125	0.402
420	0.141	-
430	0.183	-
440	0.270	-

Figure S1. Crystallographic packing of **Mn-AdDB** (**v486**) showing the nanochannels. The MOFs obtained after metal cation exchange have a similar packing.

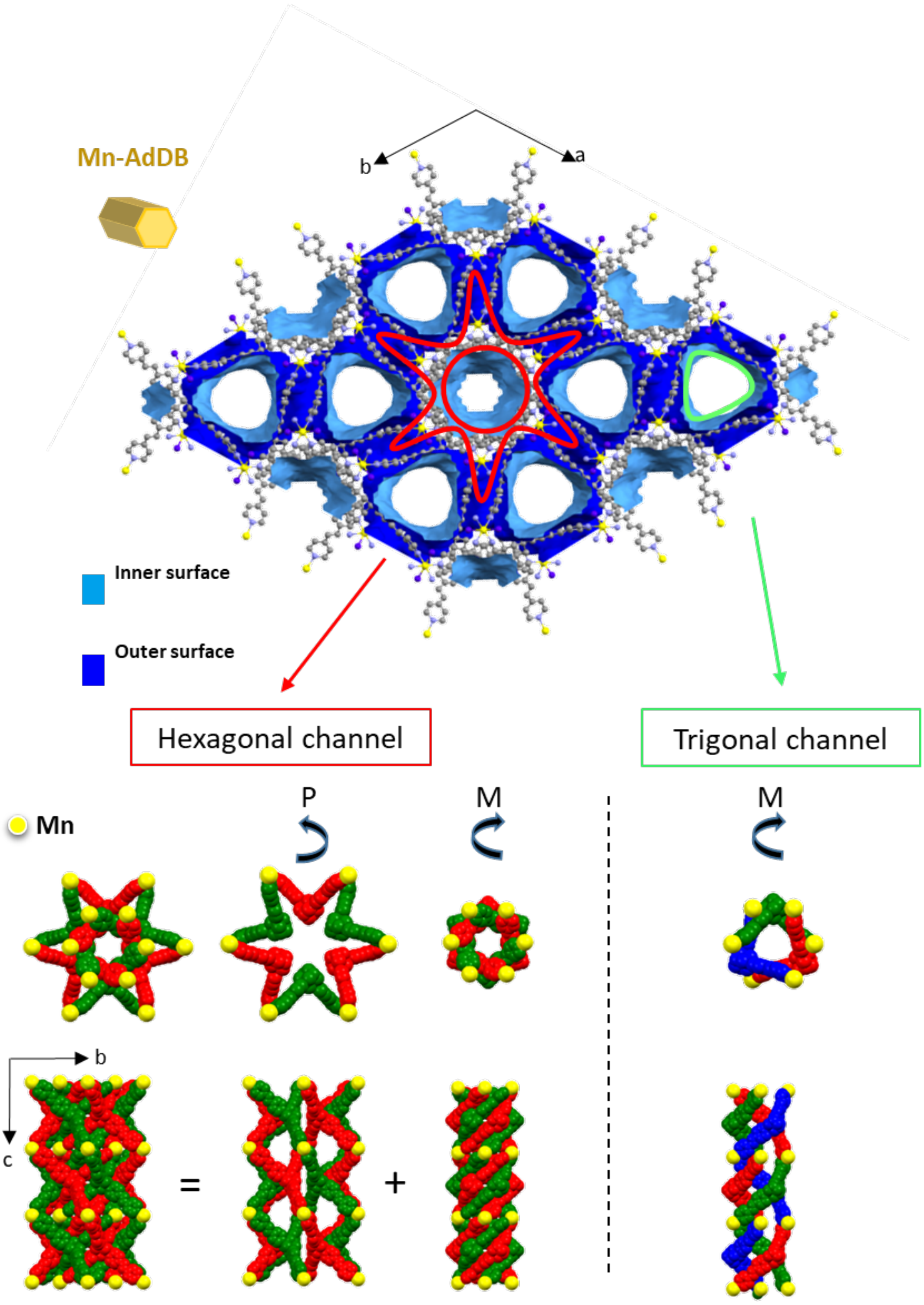

Figure S2. Optical images of isolated MOFs obtained from **Mn-AdDB** (Figure 1) after metal cation exchange.

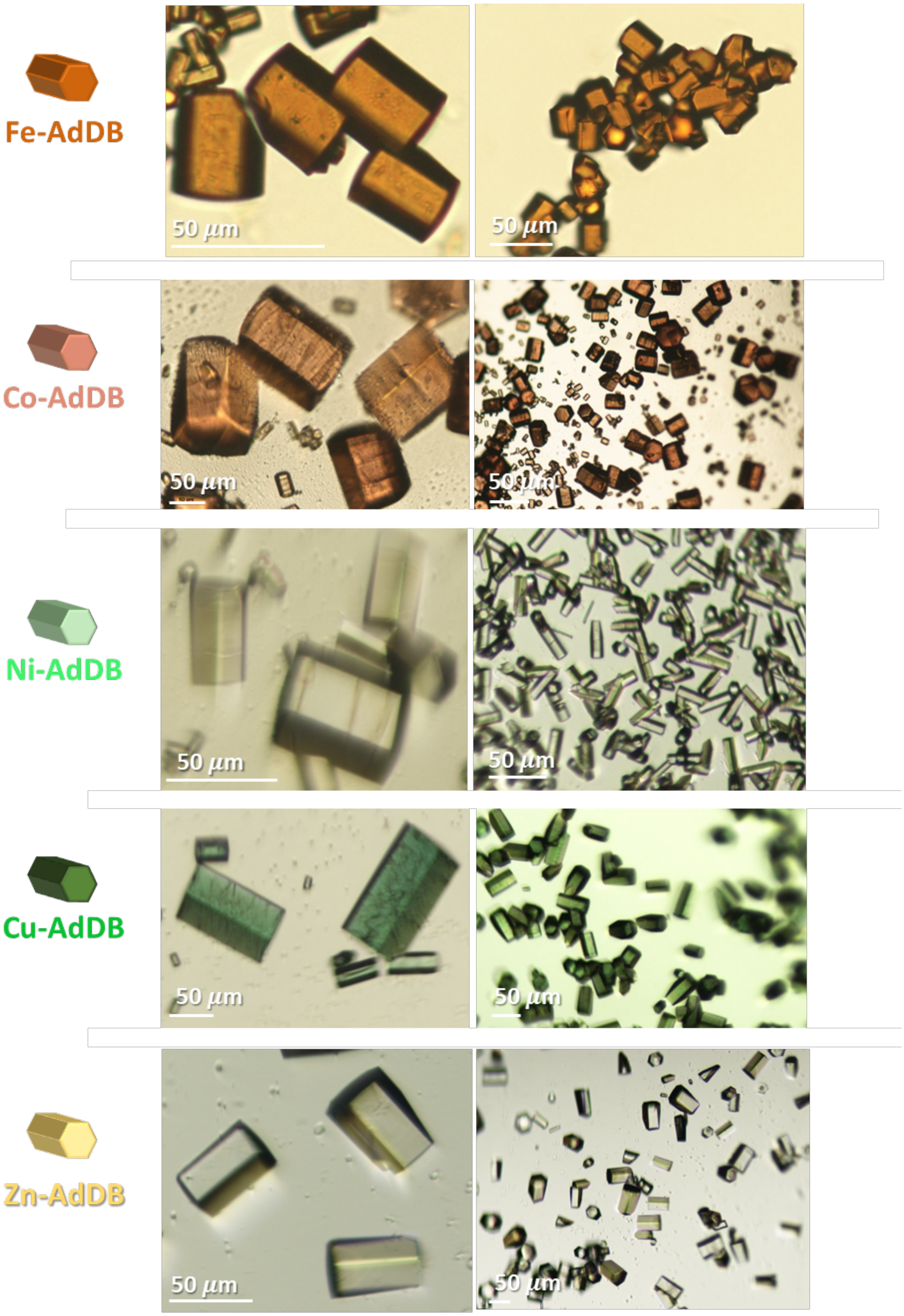

Figure S3. Scanning electron microscope (SEM) images of **M-AdDB** (M = Mn, Fe, Co, Ni, Cu, and Zn) obtained by metal cation exchange.

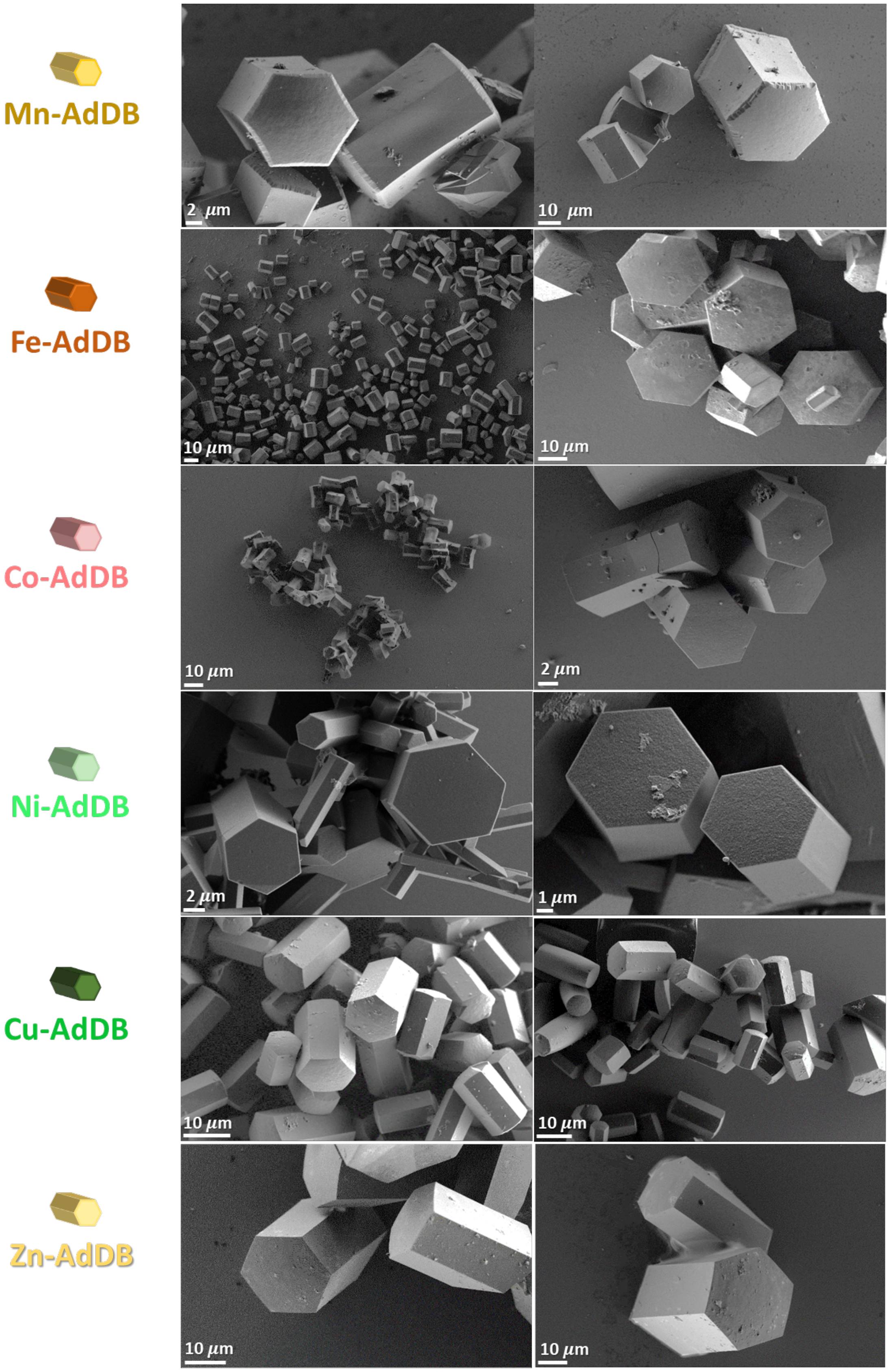

Figure S4. Experimental powder X-ray diffraction (PXRD) spectra **of Mn-AdDB** and the corresponding MOFs after metal cation exchange (black lines). The purple lines denote fits obtained using the single crystal X-ray data. Experimental intensity variations, due to the preferred orientation of the crystals on the surface, were considered in the fit by using spherical harmonic functions.[S4] The differences between the experimental intensity variations and their fits are denoted by brown lines. The goodness-of-fits are as follows: **Mn-AdDB** = 1.04; **Fe-AdDB** = 1.39; **Co-AdDB** = 1.82; **Ni-AdDB** = 1.42 and **Cu-AdDB** = 1.16.The values (a = b, c) are the estimated unit cell dimensions. **Zn-AdDB** was not sufficiently stable to measure the PXRD.

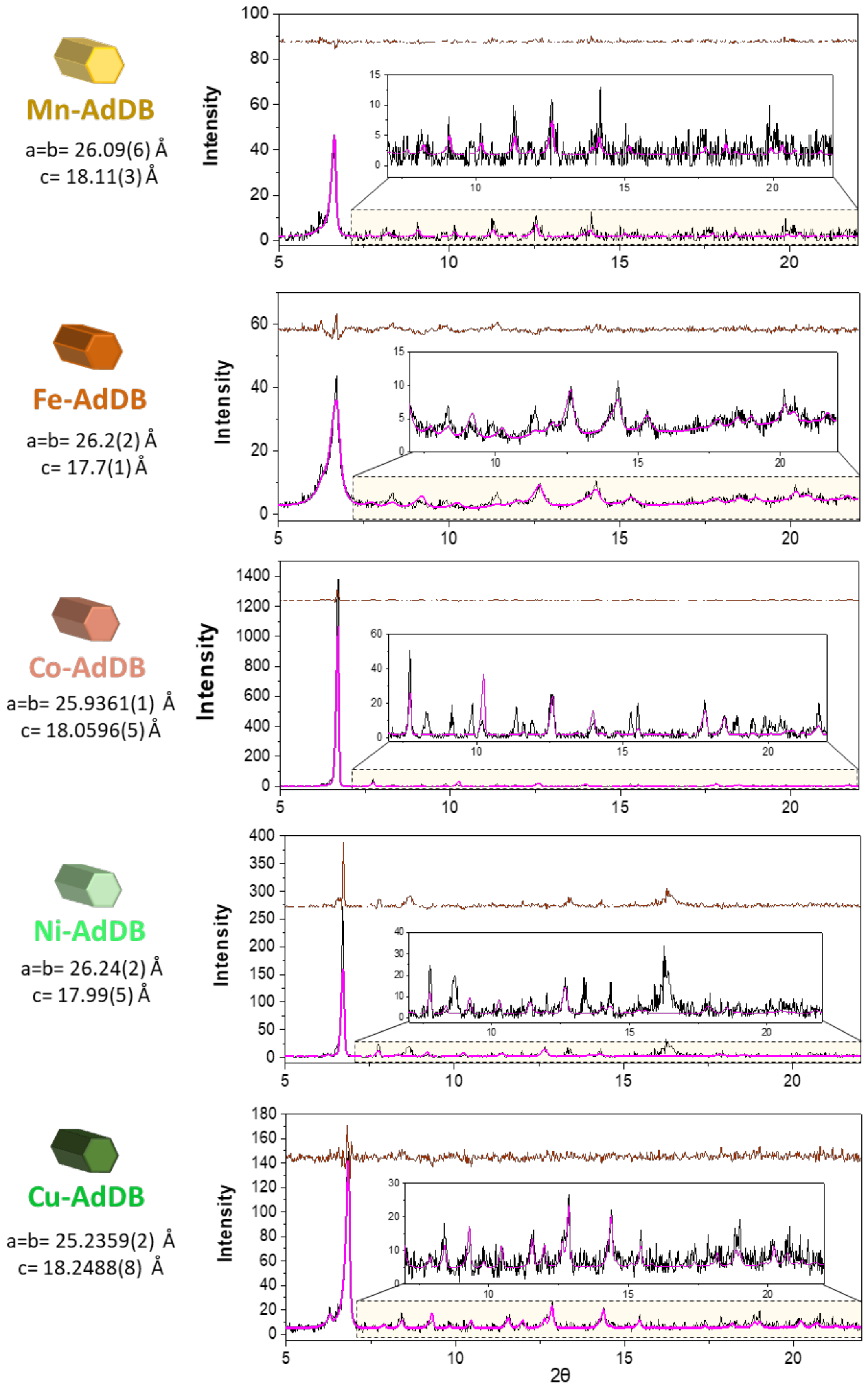

Figure S5. High-resolution X-ray Photoelectron (XPS) spectra for **Fe-AdDB** (orange lines) and **Mn-AdDB** (yellow line). Left: **Fe-AdDB**, typical doublet observed in the Fe 2p region; the peak positions are characteristic of Fe^{3+}. Right: **Mn-AdDB**, the doublet observed in the Mn 2p region is characteristic of Mn^{2+}. **Fe-AdDB**: no signal is observed, indicative of the presence of residual Mn^{2+}. The signals of Fe^{3+} and Mn^{2+} are accompanied by satellite peaks.

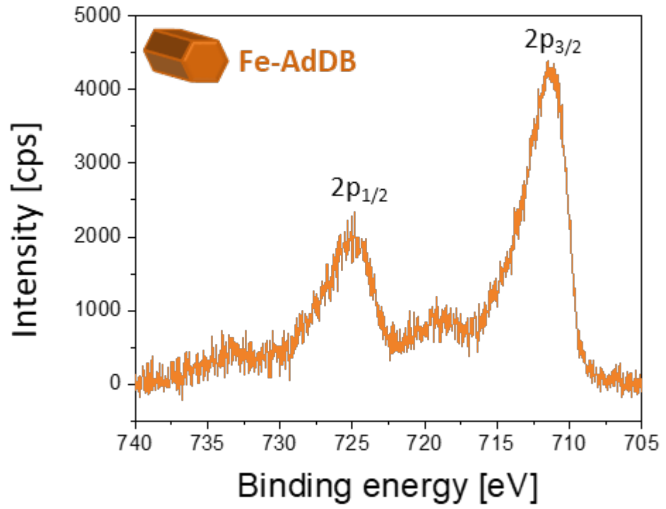

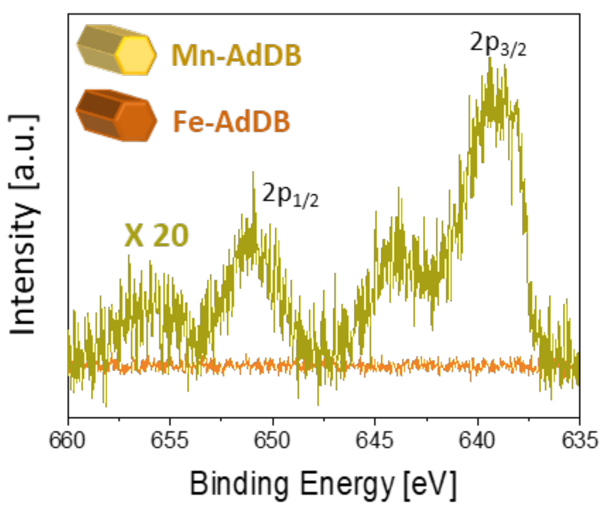

Figure S6. *In-situ* light microscopy images showing the formation of **Cu-AdDB** from **Mn-AdDB**. The crystals were reacted in a methanol solution of $CuCl_2$. Scale bar = 50 μm.

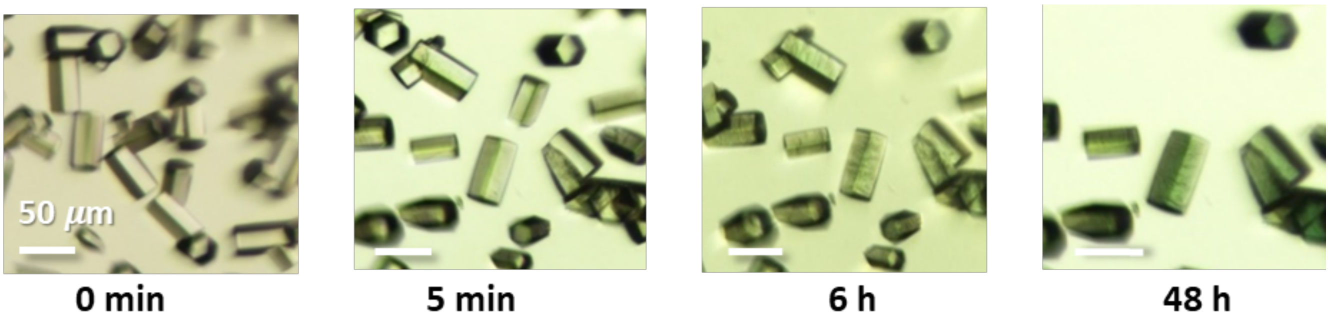

Figure S7. Top: Scanning electron microscope (SEM) images of **Fe-AdDB**. Bottom: Optical images of **Fe-AdDB** (left), **Cu-AdDB** (center), and **Co-AdDB** (right). These optical images were taken from the MOFs in the reaction mixture.

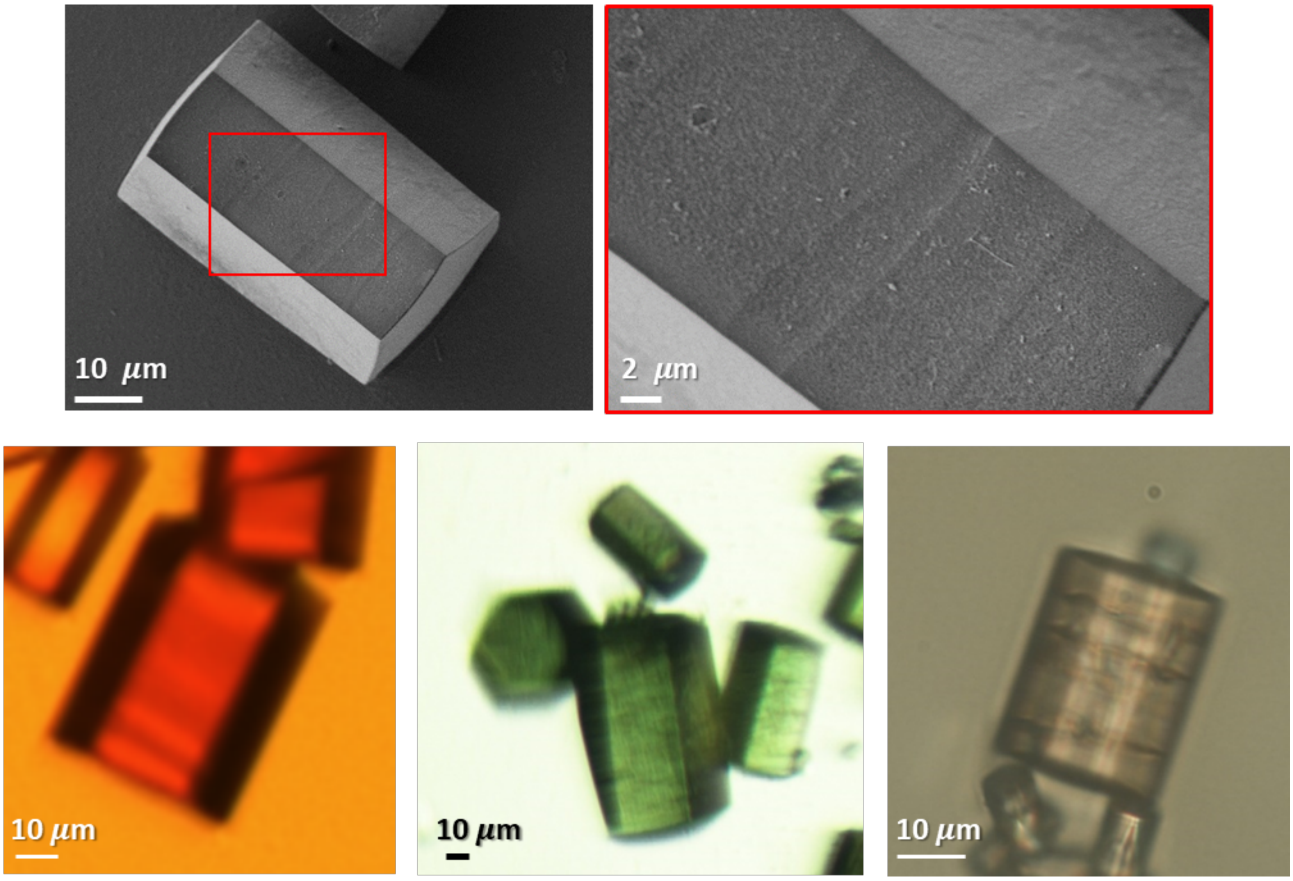

Figure S8. SEM-cathodoluminescence of single crystals. Scale bar = 2 μm.

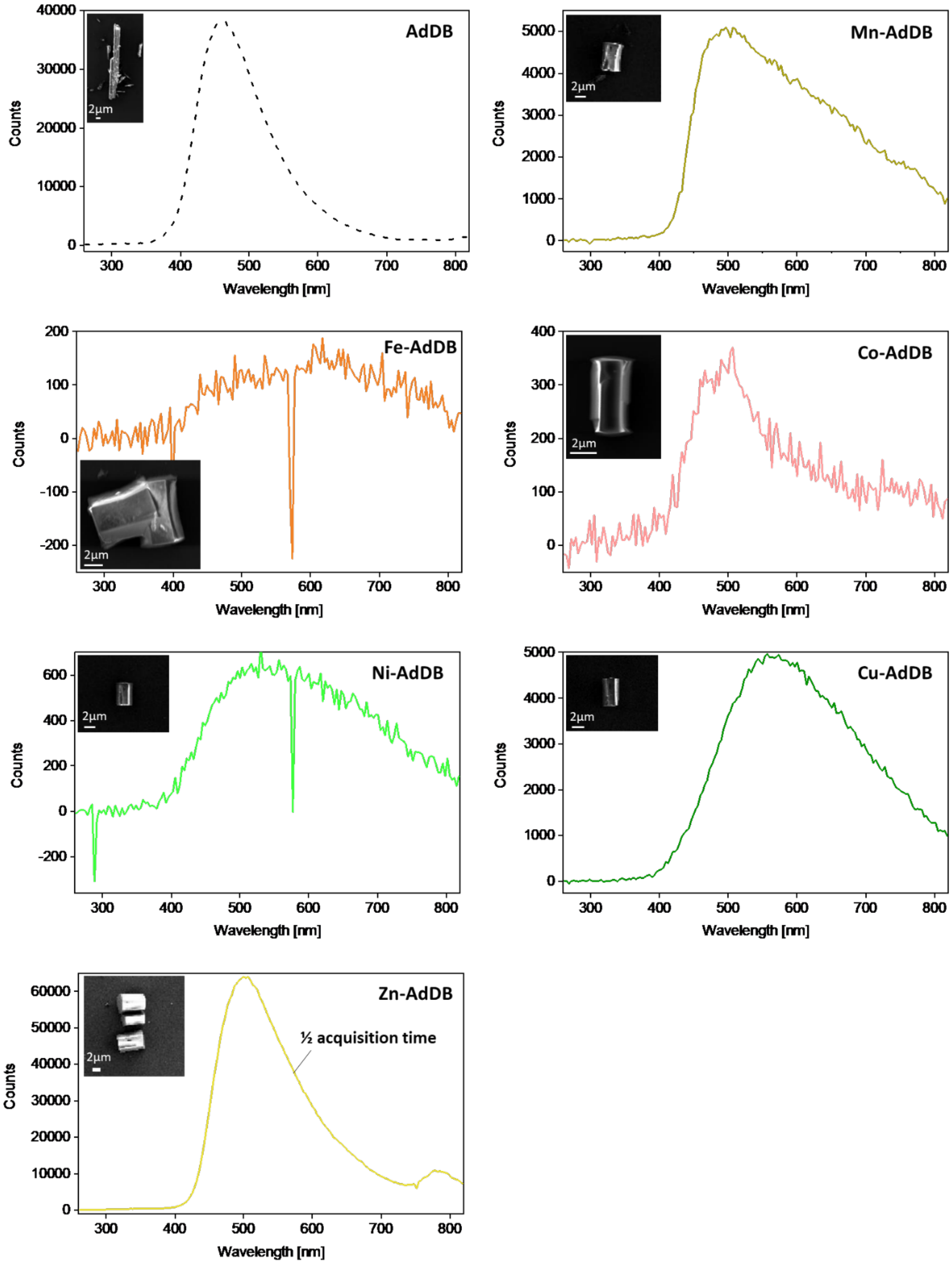

Figure S9. Lifetime measurements using a λ = 500 nm emission wavelength for **AdDB** (magenta), **Mn-AdDB** (yellow), and **Zn-AdDB** (green). The data were fitted by a mono exponential decay, deconvoluted from the system response function (RSF), shown by blue markers.

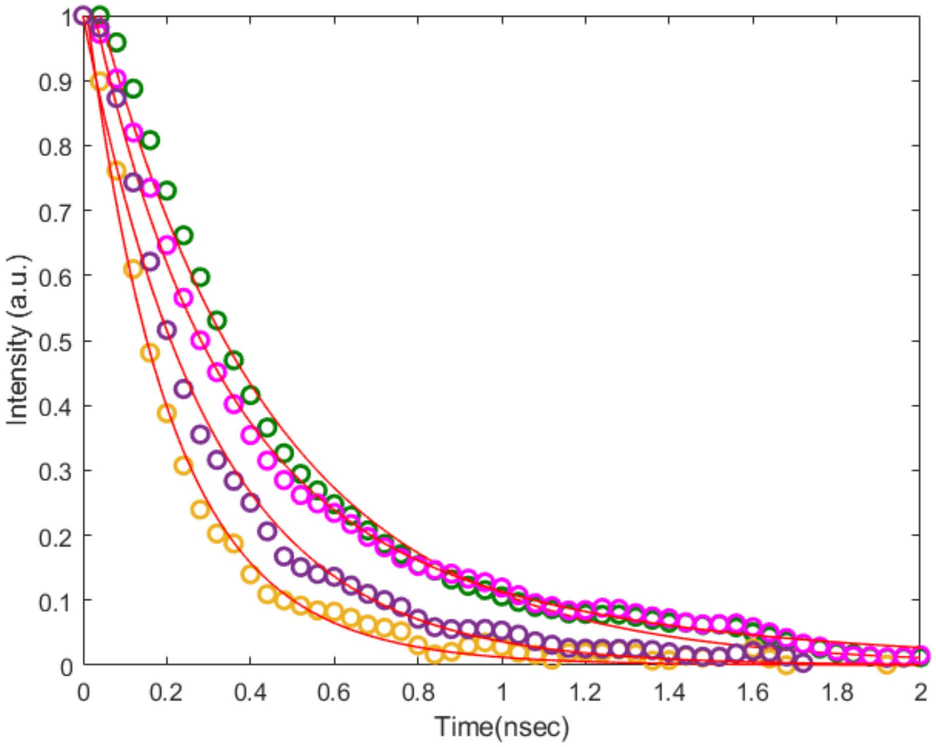

Figure S10. Plots of magnetic moment vs magnetic fields at 300K (A) and 5K (B). The magnetic susceptibility is derived from the slopes.

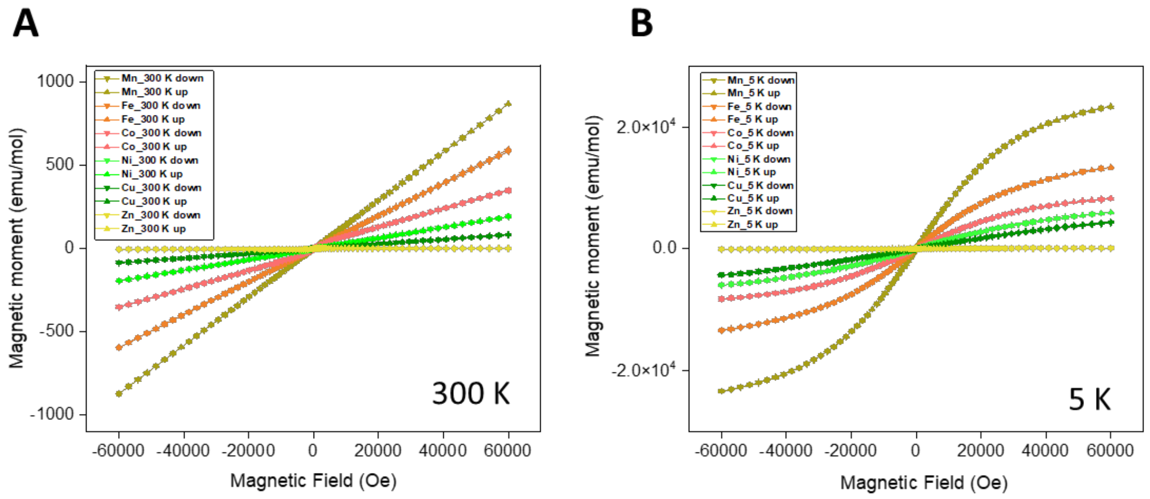

References

[S1] G. M. Sheldrick, *Acta Crystallogr. Sect. A Found. Crystallogr.* **2015**, *71*, 3–8.
[S2] O. V. Dolomanov, L. J. Bourhis, R. J. Gildea, J. A. K. Howard, H. Puschmann, *J. Appl. Crystallogr.* **2009**, *42*, 339–341.
[S3] A. L. Spek, *Acta Crystallogr. Sect. C Struct. Chem.* **2015**, *71*, 9–18.
[S4] M. Jarvinen, *J. Appl. Crystallogr.* **1993**, *26*, 525–531.
[S5] M. Vasylyev, R. Popovitz-Biro, L. J. W. Shimon, R. Neumann, *J. Mol. Struct.* **2003**, *656*, 27–35
[S6] K. Cho, J. Yoo, H. W. Noh, S. M. Lee, H. J. Kim, Y. J. Ko, H. Y. Jang, S. U. Son, *J. Mater. Chem. A.* **2017**, *5*, 8922–8926.
